# Reproducible high-quality perovskite single crystals by flux-regulated crystallization with a feedback loop

**DOI:** 10.1038/s44160-024-00576-8

**Published:** 2024-06-18

**Authors:** Yuki Haruta, Hanyang Ye, Paul Huber, Nicholas Sandor, Antoine Pavesic Junior, Sergey Dayneko, Shuang Qiu, Vishal Yeddu, Makhsud I. Saidaminov

**Affiliations:** 1 Department of Chemistry, University of Victoria, Victoria, British Columbia Canada; 2 Department of Electrical & Computer Engineering, University of Victoria, Victoria, British Columbia Canada; 3 Centre for Advanced Materials and Related Technologies (CAMTEC), University of Victoria, Victoria, British Columbia Canada

**Keywords:** Design, synthesis and processing, Design, synthesis and processing

## Abstract

Controlling the linear growth rate, a critical factor that determines crystal quality, has been a challenge in solution-grown single crystals due to complex crystallization kinetics influenced by multiple parameters. Here we introduce a flux-regulated crystallization (FRC) method to directly monitor and feedback-control the linear growth rate, circumventing the need to control individual growth conditions. When applied to metal halide perovskites, the FRC maintains a stable linear growth rate for over 40 h in synthesizing CH_3_NH_3_PbBr_3_ and CsPbBr_3_ single crystals, achieving outstanding crystallinity (quantified by a full width at half-maximum of 15.3 arcsec in the X-ray rocking curve) in a centimetre-scale single crystal. The FRC is a reliable platform for synthesizing high-quality crystals essential for commercialization and systematically exploring crystallization conditions, maintaining a key parameter—the linear growth rate—constant, which enables a comprehensive understanding of the impact of other influencing factors.

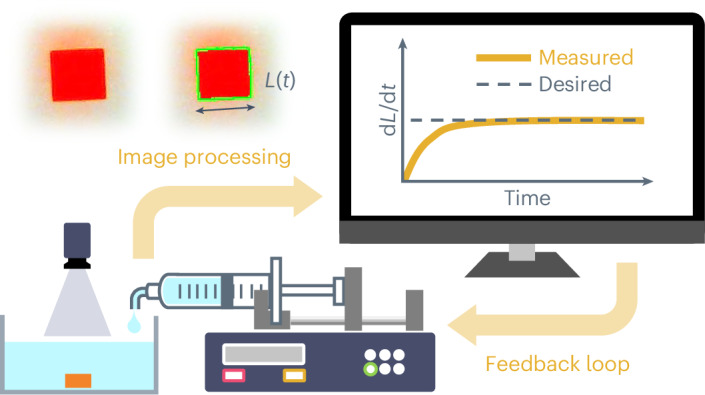

## Abstract (web summary)

Controlling linear growth rate is challenging in solution-grown single crystals. Now, flux-regulated crystallization (FRC) is developed to directly feedback-control the growth rate. When applied to metal halide perovskites, FRC achieves reproducible high crystallinity, offering a platform for synthesizing high-quality single crystals and exploring crystallization conditions.

## Main

At the fundamental research phase, access to high-quality crystals is crucial to uncover a material’s intrinsic properties because structural defects within the crystal can alter material properties. Furthermore, these defects often represent substantial barriers in the application phase of the material. Therefore, the emergence of techniques for synthesizing high-quality single crystals is often followed by the material’s successful commercialization. This is exemplified by the achievements in crystalline silicon and III–V semiconductor technology^1^.

**Fig. 1:**
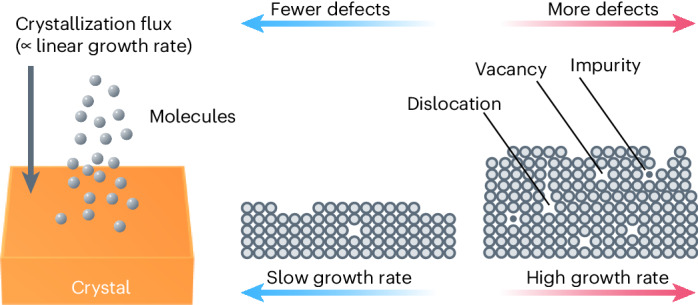
Relationship between growth rate and structural defects. Schematic demonstrating the effect of the crystallization flux (linear growth rate) on defect formation in the crystal.

Synthesis of high-quality single crystals demands careful control of a variety of parameters, including material purity, growth temperature, atmosphere and vibration, because these determine the crystallization kinetics and ultimately the quality of the obtained crystal^2^. Here, the crystal growth rate (the linear growth rate is the thickness of material deposited in a unit of time (m s^−1^)) is directly proportional to the crystallization flux (the number of molecules deposited on a unit area in a unit of time (mol m^−2^ s^−1^)) (Supplementary Note 1), and thus represents the crystallization kinetics. Generally, a slower growth rate leads to fewer structural defects because depositing molecules have enough time to settle down in the correct crystal sites, while a faster growth rate leads to an increased number of structural defects (Fig. 1)^3^. For instance, the linear growth rate of III–V semiconductors is known to notably affect the crystal quality; therefore, it is carefully controlled during crystal growth processes such as molecular beam epitaxy or metal–organic chemical vapour deposition by a feedback control system, which uses reflection high-energy electron diffraction to monitor the growth rate in situ^4,5^. In the case of crystallization from melts (for example, the Czochralski or Bridgman methods), the pulling rate from the melt and the temperature gradient determine the linear growth rate^6,7^.

In contrast to these dry processes, control over the linear growth rate in solution processes has not been well established. The most successful example is the fabrication of KH_2_PO_4_ (KDP) single crystals. KDP crystals are relatively straightforward to grow from solution because they allow an exceptionally high supersaturation level of 83% without any nucleation^8^. Although a stable linear growth rate of KDP is achieved through control of its supersaturation degree^8^, even for the easily grown KDP crystals, it requires laborious optimization with a sophisticated growth system, which includes various sensors, controllers and circulators to maintain its concentration and temperature; it is hence reasonable to assume that control of growth rate of other materials is even more challenging. For instance, metal halide perovskites are currently being extensively studied due to their high photon absorption coefficients and long charge-carrier transport^9–11^. Various perovskite single-crystal growth methods including solution cooling^12,13^, solvent evaporation^14,15^, inverse temperature crystallization (ITC)^16–19^, anti-solvent vapour-assisted crystallization (AVC)^20^ and liquid-diffused separation-induced crystallization^21,22^ have been proposed. Among these, ITC and AVC have received notable attention as they facilitate the synthesis of relatively large single crystals. However, despite efforts to address the challenges these methods present, such as their need for high temperatures and anti-solvent vapour control^23–26^, no method has been able to control the linear growth rate, and therefore its effect on crystal quality remains unexplored.

Precise control of growth conditions has been considered essential to obtain stable growth rates, and feedback control of concentration and temperature has been proposed^27–29^. However, this strategy, which can be categorized as indirect control, relies on a sophisticated system that can derive a correlation between a monitored parameter and the growth rate. If the system is strongly affected by uncertain parameters, the growth rate cannot be reproducibly regulated even if the monitored parameter is maintained as desired. Therefore, it is more effective to control the growth rate itself, that is, direct control, as already used in some dry processes. During preparation of this paper, a similar concept was applied to the growth of potassium alum by Shiele et al.^30^. A feedback system was applied to regulate the growth rate of an anchored crystal which served as an indicator for controlling growth conditions of suspended target crystals in the same solution. However, the work acknowledged that controlling of the indicator crystal does not necessarily ensure the quality of the target crystals because different growth mechanisms can coexist within the same solution^31^, and did not study the correlation between linear growth rate and crystal quality. Moreover, the longest growth time was only 6 h, resulting in target crystal sizes remaining <1 mm.

In this work, we show a flux-regulated crystallization (FRC) method that directly monitors and feedback-controls the linear growth rate of solution-grown crystals. The FRC system is established by combining an in situ image-processing and a solvent-evaporation method with a proportional-integral-derivative (PID) controller. As a case study, we mainly show the FRC of methylammonium lead tribromide (MAPbBr_3_) perovskite single crystal. The FRC allows the synthesis of centimetre-scale single crystals while maintaining a constant linear growth rate for over 40 h. Using the FRC system, we reveal the effect of the linear growth rate on the crystallinity, quantified by the full width at half-maximum (FWHM) of the (100) plane X-ray diffraction rocking curve of the crystal: the MAPbBr_3_ crystals grown at growth rates of <0.3 mm h^−1^ show high and reproducible crystallinity with the best FWHM of 15.3 arcsec in a 9.5 × 9.3 × 2.3 mm^3^ crystal at a growth rate of ~0.2 mm h^−1^, an exceptional value among reported MAPbBr_3_ single crystals. Our FRC offers a way to control the quality of single crystals, emphasizing the defining role of controlling linear growth rate in crystallization from solution.

## Results and discussion

### FRC with feedback loop

**Fig. 2:**
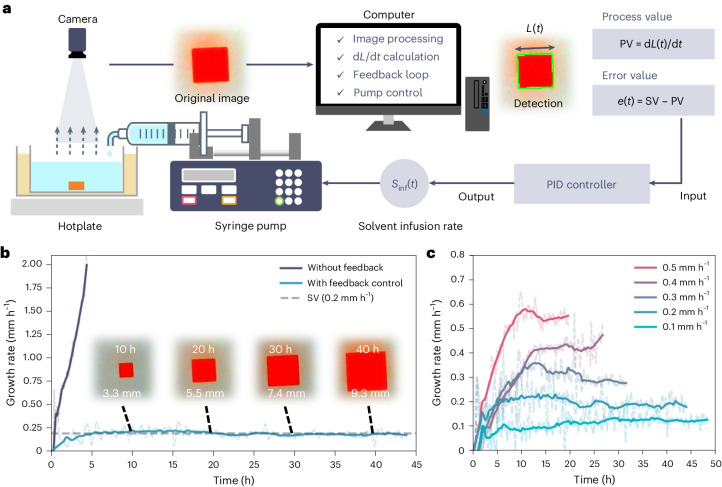
The FRC system. **a**, Schematic of the FRC system. The image of the crystal is obtained with a camera and the crystal is detected by computer vision where the green line indicates the contour of the detected region. **b**, The linear growth rates of the seed crystals with and without feedback control. Inset: images of the crystal captured at *t* = 10, 20, 30 and 40 h during SV-0.2 FRC. **c**, The linear growth rate during the FRC at SV = 0.1–0.5 mm h^−1^. The dashed and solid lines indicate the measured and smoothed growth rate, respectively. Source data

The FRC method was developed by combining a solvent-evaporation method and in situ image processing with a feedback control. The FRC system consists of three modules: a crystallization module (dish and hotplate), an image-processing module (camera and computer) and an actuator module (programmable syringe pump) (Fig. 2a and Supplementary Fig. 1). A precursor solution was prepared by dissolving 41 wt% of MAPbBr_3_ crystals in *N*,*N*-dimethylformamide (DMF). The solution was poured into the crystallization dish with a seed crystal (see Methods for seed preparation), and the dish was placed on a hotplate with an oil bath so that the solution temperature stabilizes at 40 °C (Supplementary Fig. 2). The initial solution concentration and the growth temperature were chosen so that the solution would be saturated when the temperature is stabilized (Supplementary Note 2 and Supplementary Fig. 3). During the crystallization process, the camera regularly pictures the crystal from the top. Because the solution is colourless and the MAPbBr_3_ crystal is orange, the crystal is detectable through image processing so its size (*L*) and its linear growth rate (d*L/*d*t*) can be determined during the growth. Note that only seed crystals verified as high quality through assessment with a polarized-light microscope were used for the crystal growth to ensure that any potential influence of seed quality on the results was minimized (Supplementary Note 3 and Supplementary Fig. 4). More experimental details are available in Methods, Supplementary Note 4 and Supplementary Fig. 5.

To control the linear growth rate, we implemented a PID controller, a feedback loop mechanism widely utilized in industry control systems^32^. The PID controller is governed by the following equations:

1 $$\begin{array}{c}e(t)={\rm{SV}}(t)-{\rm{PV}}(t)\end{array}$$

2 $$\begin{array}{c}u(t)={K}_{{\rm{P}}}e(t)+{K}_{{\rm{I}}}\displaystyle{\int }_{0}^{t}e(\tau ){\mathrm{d}}\tau +{K}_{{\rm{D}}}\frac{{\mathrm{d}}e(t)}{{\mathrm{d}}t}\end{array}$$

where *e*(*t*) is an error value, reflecting the difference between the desired set value (SV(*t*)) and the measured process value (PV(*t*)), *u*(*t*) is a control variable, and *K*_P_, *K*_I_ and *K*_D_ are proportional, integral and derivative coefficients. In the FRC system, SV and PV are the target linear growth rate and the measured growth rate, respectively.

Because evaporation is the driving force of crystallization in this system, it is natural to consider the evaporation rate as the control variable, *u*(*t*), to get the desired linear growth rate. The evaporation rate can be controlled by temperature or solution surface area, but the temperature also affects the solubility, making the system complex, and changing solution surface area during crystallization is similarly challenging. Hence, this system controls the net evaporation rate (*E*_net_) instead of the actual evaporation rate by adding a fresh solvent (DMF) via a syringe pump. We assumed that the actual evaporation rate (*E*_act_) is constant because the temperature is constant during the crystallization, and then defined the *E*_net_ as the control variable by the following equation:

3 $$\begin{array}{c}u(t):{E}_{{\rm{net}}}(t)={E}_{{\rm{act}}}-{S}_{\inf }(t)\end{array}$$

where the *S*_inf_ is the solvent infusion rate. Combining equations (2) and (3), we obtain the control equation:

4 $$\begin{array}{c}{S}_{{{\inf }}}(t)={E}_{{\rm{act}}}-{K}_{{\rm{P}}}e(t)-{K}_{{\rm{I}}}\displaystyle{\int }_{0}^{t}e(\tau ){\mathrm{d}}\tau -{K}_{{\rm{D}}}\frac{{\mathrm{d}}e(t)}{{\mathrm{d}}t}\end{array}$$

Because *E*_act_ is not known until the experiment concludes, we utilized the estimated evaporation rate (*E*_est_) instead, leading to the following control equation for the FRC:

5 $$\begin{array}{c}{S}_{{{\inf }}}(t)={E}_{{\rm{est}}}-{K}_{{\rm{P}}}e(t)-{K}_{{\rm{I}}}\displaystyle{\int }_{0}^{t}e(\tau ){\mathrm{d}}\tau -{K}_{{\rm{D}}}\frac{{\mathrm{d}}e(t)}{{\mathrm{d}}t}\end{array}$$

The *E*_est_ value is a preset constant based on previous values of *E*_act_, which were obtained from the mass of the syringes and the container before and after the crystal growth. Note that the *E*_act_ values ranged between 2.08 and 3.60 g h^−1^ (*n* = 77; median, 2.66 g h^−1^), despite our efforts to maintain consistent experimental conditions (Supplementary Fig. 6).

Figure 2b and Supplementary Movie 1 show representative growth rate curves when the seed crystal was grown with the feedback control (FRC) and without it (only solvent evaporation). The growth rate was determined as a slope of the linear regression applied to the preceding 10 min of *L*(*t*) data (see Supplementary Note 4 for details). The growth rate was smoothed to eliminate the noise caused by image-processing errors. The validity of the data smoothing was confirmed in Supplementary Note 5 and Supplementary Figs. 7–9. Without feedback control, the crystal growth rate steeply increased and reached nearly 2.0 mm h^−1^ within 4 h. This result indicates that the supersaturation level continuously increased because solvent evaporation caused a greater rise in concentration than the reduction from crystal growth. The steep increase also caused a lot of nucleation, making it difficult to obtain a large crystal (Supplementary Fig. 10). On the contrary, in the case of the FRC, the growth rate ceased rising upon nearing SV = 0.2 mm h^−1^ and remained stable at this level for over 40 h. This sustained crystal growth stability is further evidenced by representative crystal images captured at *t* = 10, 20, 30 and 40 h during the FRC (Fig. 2b, inset), revealing average growth rates of 0.22, 0.19 and 0.19 mm h^−1^ for each successive 10 h interval. Furthermore, the FRC at the SV of 0.2 mm h^−1^ (SV-0.2) exhibited only few or occasionally no nucleation for over 45 h (Supplementary Fig. 11). The FRC system could also control the growth rate at SV values of 0.1–0.5 mm h^−1^ (Fig. 2c). The control delay in the FRC system (the time for the control variable to influence the objective variable) was estimated to be 23–48 min (Supplementary Note 6). The control equation (equation 5) displayed effective responsiveness in addressing this delay (Supplementary Fig. 12).

### Structural characterization of crystals grown by FRC

**Fig. 3:**
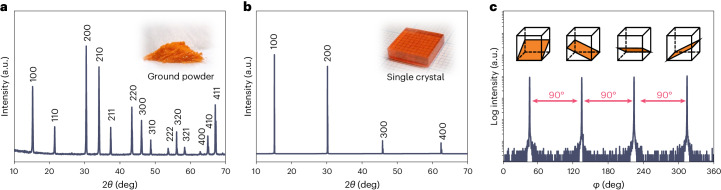
X-ray diffraction of the MAPbBr_3_ crystals produced by FRC. **a**,**b**, X-ray diffraction 2*θ*/*ω* scan of the ground powder (**a**) and the obtained crystal (**b**). Inset: images of the samples. **c**, *φ* scan of MAPbBr_3_ single crystal to detect the {210} diffraction peaks. Inset: the {210} planes in a cubic unit cell with a quadruple symmetry. Source data

Figure 3a shows X-ray diffraction 2*θ*/*ω* scan of ground FRC crystals: all observed peaks matched precisely with the expected peaks from cubic MAPbBr_3_. Intact crystals diffract only from the (100), (200), (300) and (400) crystallographic planes (Fig. 3b), indicating that the exposed facets of the rectangular crystal (Fig. 3b, inset) correspond to the {100} crystallographic plane, which is consistent with previous studies^33^. We also performed a phi (*φ*) scan where 2*θ* and chi (*χ*) were fixed at 34.06° and 26.57°, respectively, to detect diffraction peaks from the {210} planes. As shown in Fig. 3c, four peaks with a 90° interval were detected, indicative of the crystal’s quadruple symmetry as one would expect from its cubic crystal structure.

### Control accuracy of the FRC system

**Fig. 4:**
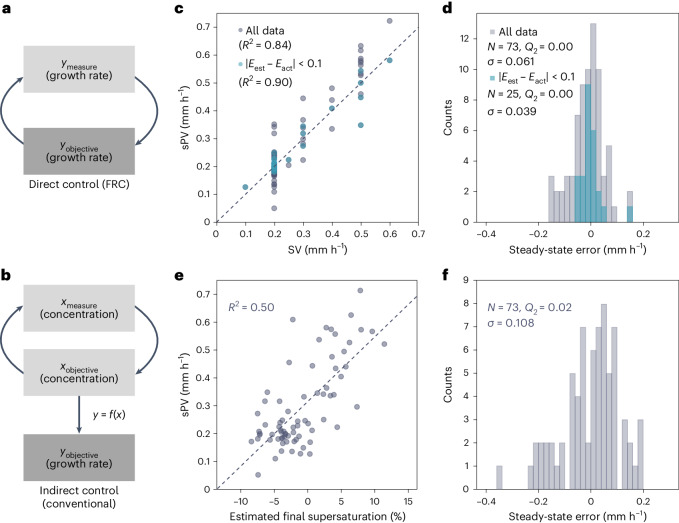
Direct and indirect control of the linear growth rate. **a**,**b**, Schematics of direct control (**a**) and indirect control (**b**). **c**, Stabilized growth rates (sPV) at varying set values (SV). The dashed line indicates an ideal control (sPV = SV). The blue dots represent the filtered data when the evaporation estimation error was <0.1 g h^−1^. **d**, Histogram of the steady-state error (SV − sPV) derived from the fitting line in **c**. The blue bins represent the filtered data when the evaporation estimation error was <0.1 g h^−1^. **e**, Values of sPV and the estimated final supersaturation level. The dashed line indicates a linear fitting. **f**, Histogram of the steady-state error derived from the gap between the data point and the fitting line in **e**. Source data

Feedback systems, as briefly mentioned above, can be categorized into two types: direct control feedback, in which the target parameter, that is, the growth rate, is directly monitored and utilized within the feedback loop (Fig. 4a); and indirect control feedback, a conventional approach for solution-grown single crystals, in which a different variable, often the concentration, is monitored to regulate the growth rate (Fig. 4b). The indirect method typically necessitates more intricate control logic to interpret the system’s internal state from indirect measurements, leading to increased control errors compared with direct control, in which adjustments are made in real-time based on the actual growth rate.

The FRC method is a direct control system. To evaluate its accuracy, we plotted the stabilized growth rate (sPV, the average PV over the last 3 h of crystal growth) against SV. The resulting coefficient of determination (*R*^2^) of 0.84, when compared to the linear regression fit (sPV = SV) expected in an ideal control scenario, indicates a robust performance of our feedback control system in maintaining the desired growth rates (Fig. 4c). This robust performance is further evidenced by the histogram of the steady-state errors (SV − sPV) that shows a median (*Q*_2_) of 0.00 mm h^−1^ and a standard deviation (*σ*) of 0.061 mm h^−1^ (Fig. 4d).

Our analysis identified a critical factor influencing the system’s control accuracy, the estimation of the evaporation rate, which the FRC system incorporates into its control equation (equation (5)). We observed a positive correlation (*r*_Pearson_ = 0.555) between the steady-state errors (SV − sPV) and the evaporation rate estimation errors (*E*_est_ − *E*_act_), as shown in Supplementary Fig. 13a. Remarkably, when filtering the data for cases with an evaporation estimation error below ±0.1 g h^−1^, we observed improved control accuracy, evidenced by a higher *R*^2^ value of 0.90 (blue dots in Fig. 4c) and a reduced *σ* of 0.039 mm h^−1^ (blue bins in Fig. 4d and Supplementary Fig. 13b), compared with other cases where *σ* = 0.069 mm h^−1^ (Supplementary Fig. 13c). These findings underscore the importance of further refining the accuracy of evaporation rate estimations, which could be accomplished in the future by deploying more sophisticated sensors or enhancing the algorithms that predict evaporation based on environmental and system variables.

For the KDP crystals discussed above, a clear linear relationship was previously found between the supersaturation level and the linear growth rate^8^, making indirect control feasible for it. To probe if that is also the case for the growth of MAPbBr_3_ single crystals, we estimated the supersaturation level during the crystal growth with the aid of image processing (Supplementary Note 7) and plotted it against the linear growth rate for experiments conducted with and without FRC (Supplementary Fig. 14). Note that the estimated supersaturation level represents an average value in the crystallizing dish, rather than local supersaturation level at the solid–solution interface, which directly affects the crystal growth. In the case of non-FRC, the growth rate increased with increasing supersaturation, exhibiting a nearly linear relationship. In the case of FRC with SV-0.2, the growth rate showed a similar trend, albeit not as strongly correlated, suggesting that relying on supersaturation–growth rate relationship for indirect control of MAPbBr_3_ growth is inaccurate.

To further support this argument statistically, we estimated the final supersaturation level for 73 experiments and plotted this against sPV, revealing a poor *R*^2^ of 0.50 (Fig. 4e). This poor control is also evidenced by the histogram of the steady-state errors (gaps from the linear fitting line) which displays a wide *σ* of 0.108 mm h^−1^ (Fig. 4f). Additionally, it is worth noting that a negative supersaturation level leading to a positive growth rate is unnatural, which we attribute to the challenges in accurately estimating the supersaturation level. The supersaturation determined by total mass measurements relies on the assumption of uniform solution concentration, which is not the case for the FRC system due to the constant supply of solvent. Therefore, achieving a better indirect control necessitates the development and application of a concentration distribution model, a formidable challenge as the concentration distribution is influenced by local events, such as temperature fluctuations, enthalpy of crystallization, solute consumption by crystal growth and nucleation, and other unpredictable factors.

### Application of the FRC system to CsPbBr_3_

We also applied the FRC system to the synthesis of CsPbBr_3_ single crystals. Compared to MABr–PbBr_2_, the CsBr–PbBr_2_ system is more complex as its phase diagram exhibits three different distinct compositions; in addition, the unbalanced solubility between CsBr and PbBr_2_ in organic solvents requires the use of an off-stoichiometric solution^34^. The precursor solution was prepared following the recipe reported by Pan et al. for the ITC method^35^ where CsBr and PbBr_2_ in a 1:2 molar ratio were dissolved in dimethylsulfoxide with tetramethyl ammonium bromide as an additive. After several experiments to optimize the initial conditions, such as the solution concentration and the temperature, we could achieve a stable growth rate of 0.1 mm h^−1^ for more than 40 h (Supplementary Fig. 15), showcasing the generalizability of the FRC system.

### Influence of linear growth rate on crystallinity

**Fig. 5:**
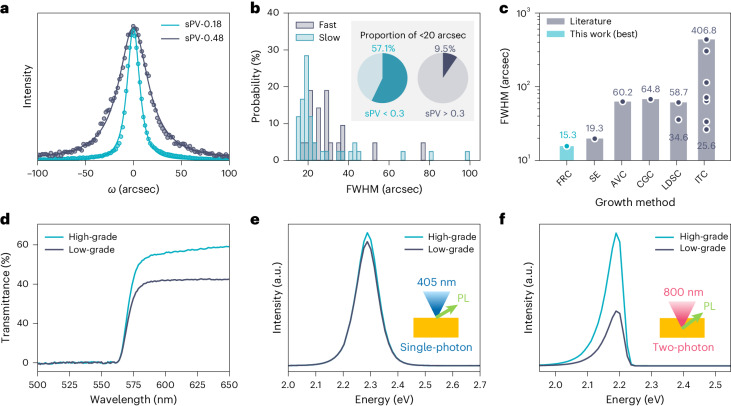
X-ray diffraction and optical properties of MAPbBr_3_ crystals. **a**, (100) rocking curves of sPV-0.18 and sPV-0.48 crystals. The dots and lines are the experimental data and fitting curve, respectively. **b**, Histogram of the FWHM of the (100) rocking curve when sPV was slower than 0.3 mm h^−1^ and faster than 0.3 mm h^−1^. Inset: proportion of the crystals with FWHM < 20 arcsec. **c**, Comparison of the FWHM with the MAPbBr_3_ single crystals grown by different methods (FRC, this work; SE, solvent evaporation^14^; AVC^25^; CGC, counterdiffusion-in-gel growth^47^; LDSC, liquid-diffused separation-induced crystallization^21,22^; ITC^22,33,44,48–50^). Note that SE^14^ and CGC^47^ used the (200) and (220) planes for the rocking-curve measurement, whereas the others use the (100) plane. **d**–**f**, Transmittance (**d**), single-photon PL (**e**) and two-photon PL (**f**) spectra of the high-grade and low-grade MAPbBr_3_ crystals. The excitation wavelengths for single-photon and two-photon PL are 405 nm and 800 nm, respectively. Insets in **e** and **f**: schematics of the PL measurement via a confocal microscope. Source data

A rocking-curve measurement (*ω* scan) was performed for the (100) crystallographic plane of the MAPbBr_3_ crystals by high-resolution X-ray diffraction. The rocking curve measures the broadening of diffraction peaks when a crystal is tilted, caused by misorientation or disordering of crystallites. Figure 5a shows representative rocking curves of FRC MAPbBr_3_ crystals grown at sPV of 0.18 and 0.48 mm h^−1^ (denoted as sPV-0.18, sPV-0.48 crystal hereafter). To quantify the crystallinity, the FWHM values were derived via a peak fitting with the Pearson-VII function (Supplementary Note 8). As shown in Fig. 5a, the Pearson-VII function accurately fitted the experimental data with high *R*^2^ > 0.99. The sPV-0.18 crystal showed a rocking curve with a FWHM of 15.3 arcsec which is narrower than that of the sPV-0.48 (FWHM, 37.4 arcsec), suggesting its well-ordered crystallinity.

We conducted rocking-curve measurements on 63 crystals, varying in sPV values, and observed a cluster with FWHM concentrated around 20 arcsec for sPV < 0.30 mm h^−1^ (referred to as slow-grown crystals), while data points were widely scattered for the fast-grown crystals with sPV > 0.30 (Supplementary Fig. 16). The FWHM histograms illustrate that the slow-grown crystals showed a median of 19.6 arcsec and a narrow quartile range of 7.5 arcsec (*n* = 42), indicating high reproducibility (Fig. 5b). In contrast, the fast-grown crystals exhibited a broader distribution, with a median of 28.1 arcsec and a quartile range of 14.0 arcsec (*n* = 21). Setting a benchmark FWHM of 20 arcsec—based on the previously documented record of 19.3 arcsec for MAPbBr_3_ crystals^14^—we observed that fast-grown crystals achieved a success rate of only 9.5%, whereas this success rate was six times higher for slow-grown crystals (Fig. 5b, inset).

Based on these results, we conclude that slow linear growth rates (<0.30 mm h^−1^) provide high crystallinity with reproducibility. The best FWHM of 15.3 arcsec was obtained from sPV-0.18 crystal with the size of 9.5 × 9.3 × 2.3 mm^3^, which is comparable to a commercial silicon wafer (15.8 arcsec, Supplementary Fig. 17) and to the record value among reported MAPbBr_3_ single crystals (Fig. 5c and Supplementary Table 1). The best crystal showed similar (100)-plane rocking curves at nine different locations with a FWHM of 15.3–17.9 arcsec, indicating its excellent uniformity (Supplementary Fig. 18). Furthermore, the rocking curves for the (200), (300) and (400) planes showed FWHMs of 14.0, 11.8 and 13.8 arcsec, respectively (Supplementary Fig. 19). We note that although the reproducibility of fast-grown crystals is not high, the occasional achievement of high crystallinity under these conditions (for example, sPV = 0.68 mm h^−1^, FWHM = 17.4 arcsec) will stimulate future work to enable the production of high-quality single crystals with high growth rates.

We noticed that visible defects are often observed from the fast-grown crystals. Supplementary Fig. 20a,b shows microscope images with and without light polarization of representative slow-grown (sPV-0.17) and fast-grown (sPV-0.57) crystals. When the polarizer was not used, a shadow was observed in the sPV-0.57 crystal, suggesting that this part scatters the light, whereas no shadow was observed in the sPV-0.17 crystal (Supplementary Fig. 20a). When the vertical polarizers were used, the shadow part corresponds to a brighter region in the dark polarized images (Supplementary Fig. 20b), suggesting the existence of structural defects. Such visible defects were observed in 12 out of 21 (57%) fast-grown obtained crystals, while this occurrence was noted in only 1 out of 14 (7%) slow-grown crystal (Supplementary Fig. 20c).

### Optical and electronic properties of crystals grown by FRC

We then measured the optical properties of MAPbBr_3_ crystals categorized into high-grade crystallinity (FWHM = 20 arcsec based on the X-ray rocking curve) and low-grade crystallinity (FWHM = 99 arcsec). Note that crystals with visible defects were excluded from this analysis. Figure 5d shows the light transmittance spectra: the high-grade crystal has a higher transmittance for the low-energy photons, indicating fewer scattering sites inside the crystal. In addition, we calculated the Urbach energy, a measure of energy disorder near the band edge (Supplementary Fig. 21): the high-grade crystal offers a low Urbach energy of 18.7 meV compared with the low-grade crystal (24.4 meV), suggesting that high crystallinity also leads to lower energy disorder in the crystals.

Both crystals showed nearly identical photoluminescence (PL) spectra when excited by a 405 nm laser (Fig. 5e). Given that the penetration depth of 405 nm light is <1 μm, the results indicate that both crystals have a similar density of defect states at the surfaces regardless of the bulk crystallinity.

We then employed two-photon absorption-induced PL (TPA-PL), widely used for probing bulk defect states in perovskite single crystals^36–41^. In the TPA process, two photons with energy below the bandgap (0.5*E*_g_ < *hν* < *E*_g_) are absorbed simultaneously, exciting an electron. Because the energy of the excitation laser is below the bandgap (1.55 eV in this study), it penetrates through the surface and is absorbed within the bulk of the crystal. Both crystals showed redshifted and asymmetric TPA-PL peaks compared with the single-photon PL, but the high-grade crystal showed notably higher intensity compared with the low-grade crystal (Fig. 5f). The observed redshift and asymmetricity in the TPA-PL spectra are often attributed to reabsorption of emitted light and the difference in the bandgap between the surface and the bulk (Supplementary Note 9 and Supplementary Fig. 22)^39,40^. Considering that the focal depth was the same and only several micrometres, the difference in the intensity cannot be attributed to reabsorption at the surface. Therefore, we infer that the low-grade crystal has more bulk defects, leading to reduced radiative recombination. Time-resolved PL measurements conducted on freshly cleaved surfaces of the crystals revealed that high-grade crystals exhibit longer PL lifetimes, averaging 345 ns, compared with low-grade crystals, which average 176 ns (Supplementary Note 10 and Supplementary Fig. 23), further supporting the findings from TPA-PL.

We also evaluated the charge carrier mobility and the mobility-to-lifetime product of the crystals in ohmic junctions (Au/MAPbBr_3_/Au) using time-of-flight measurement and analysing their response to visible light, and by determining the X-ray detection properties in Schottky junctions (Au/MAPbBr_3_/Ga) (Supplementary Note 11 and Supplementary Figs. 24–26). Overall, the slow-grown crystals showed improved performance, with occasional instances of exceptional properties, such as a high carrier mobility of 217 cm^2^ V^−1^ s^−1^. However, we refrain from positioning the electrical measurements as a definitive method for crystal evaluation because perovskites are known to be sensitive to factors such as ion migration^42^, surface defects^43,44^ and interface electrochemical reactions^45^, which can strongly influence electrical measurements. Irreproducibility in perovskite device fabrication is also known to notably alter device performance, including dark current and photoresponse^35^. For perovskite materials, a more detailed study of the relationship between crystallinity and electrical properties is required, along with the development of a reproducible device fabrication process.

## Conclusion

We developed a FRC system capable of maintaining stable growth for over 40 h in the growth of MAPbBr_3_ and CsPbBr_3_ single crystals. We determined that a deliberately slow growth, <0.3 mm h^−1^, is crucial to secure both high reproducibility and high crystallinity in the synthesis of MAPbBr_3_ single crystals. The achieved exceptionally high crystallinity (FWHM = 15.3 arcsec) opens new applications for MAPbBr_3_, such as its potential use in an X-ray mirror or monochromator—a prospect we are currently investigating. While our adopted image-processing algorithm presently confines FRC to processes exhibiting a clear contrast between the crystal and the solution, we acknowledge that this limitation can be overcome (and the FRC approach generalized) through the development of advanced imaging techniques. We anticipate that FRC will contribute to commercialization of perovskite crystals as a reliable synthesis process. We also posit that FRC stands as a robust tool for advancing research in crystallization dynamics: its application allows for a nuanced exploration of the influence of various growth conditions, such as temperature and additives, by enabling direct comparisons between identical growth rates.

## Methods

### Reagents

Methylammonium bromide (MABr, 99.999%) was purchased from Greatcell Solar Materials. Lead bromide (PbBr_2_, >98%) was purchased from Sigma-Aldrich. DMF and sulfuric acid (93–98%) were purchased from Fisher Chemical. Dimethoxydimethylsilane and cyclohexane were purchased from Tokyo Chemical Industry. Isopropanol (99.5%) was purchased from VWR chemicals.

### Hydrophobic treatments

Hydrophobic treatment was performed for all dishes and containers that were used for crystallization to suppress undesirable nucleation. For the treatment, we followed the method reported in a previous report^46^: 200 ml of isopropanol, 17.5 ml of dimethoxydimethylsilane and 0.8 ml of sulfuric acid were mixed and left for 30 min at room temperature before use. Prior to performing the hydrophobic treatment, the dishes were cleaned with water and isopropanol to remove dust. Then the reaction solution was poured into the dish. One minute later, the solution was removed and the dish was cleaned with isopropanol.

### Recrystallization of MAPbBr_3_ crystals for precursors

A MAPbBr_3_ solution is prepared by dissolving MABr and PbBr_2_ at an equivalent molar ratio in DMF with a concentration of approximately 40 wt%, that is, 9.4 g MABr, 30.7 g PbBr_2_, and 60.0 g DMF. The solution was kept stirring overnight at room temperature for complete dissolution. The solution was poured into a 500 ml beaker and the beaker was placed on a 50 °C hotplate. After several days, a decent amount of MAPbBr_3_ crystals were deposited in the solution because of solvent evaporation. The crystals were collected, the remaining solution was wiped away, and the crystals were washed with cyclohexane and then were dried in a vacuum overnight.

### Preparation of seed crystals

A 41 wt% MAPbBr_3_ solution was prepared by dissolving the MAPbBr_3_ crystals in DMF and stirring overnight. Then, 100 g of the solution was poured into a crystallization dish (diameter, 100 mm) after filtration with a 0.22 µm PTFE filter and the dish was placed on a hotplate with an oil bath. The hotplate temperature was raised up to 50 °C and kept at this temperature. After a few hours, several orange crystals were deposited in the solution. Well-shaped crystals were collected, the remaining solution was wiped away, and these crystals were washed with cyclohexane and dried in a vacuum overnight. The obtained crystals were also observed under a polarized microscope to select the seeds (Supplementary Fig. 4).

### Single-crystal growth

A 41 wt% MAPbBr_3_ solution was prepared by dissolving the MAPbBr_3_ crystals in DMF and stirring overnight. Then, 100 g of the solution was poured into a crystallization dish (diameter, 100 mm) after filtration with a 0.22 µm PTFE filter. A hydrophobically treated cover slip was placed in the dish to create a flat bottom. The dish was placed on a hotplate with an oil bath. After the seed crystal (<2 mm) had been immersed in the solution, the hotplate temperature was heated so that the solution temperature at the bottom centre stabilized at 40 °C. The code to operate the FRC system is described in Supplementary Note 4. Supplementary Movie 2 shows the image processing during crystal growth. After the crystallization, the obtained crystals were collected, the remaining solution was wiped away, and the crystals were washed with cyclohexane and then dried.

### Characterization of the single crystals

X-ray diffraction 2*θ*/*ω* scans and *φ* scans were done with a PANalytical Empyrean diffractometer using a copper X-ray source. The accelerating voltage and tube currents were 45 kV and 40 mA, respectively. The rocking-curve measurements were obtained using a four-crystal Ge(220) monochromator set for Cu Kα_1_ radiation (*λ* = 1.54059 Å). The accelerating voltage and tube currents were 40 kV and 20 mA, respectively, and the step size was 0.0005° (1.8 arcsec). The transmittance and absorbance spectra were measured using a PerkinElmer Lambda 1050. The PL spectra were measured using a Carl Zeiss LSM800 confocal microscope.

## Supplementary information

Supplementary Information Supplementary Figs. 1–26, Notes 1–11 and Table 1.

Supplementary Video 1 A video of crystallization with and without feedback control.

Supplementary Video 2 A video of crystallization before and after image processing.

## Source data

Source Data Fig. 2 Growth rate source data.

Source Data Fig. 3 X-ray diffraction source data.

Source Data Fig. 4 Growth rate statistical source data.

Source Data Fig. 5 Rocking curve and photoluminescence source data.

## Data Availability

All of the data supporting the findings of this study are available in the article and its Supplementary Information.

## Code availability

The code for this study is available at https://github.com/Yuki-Haruta/2024NatureSynthesis-FRC.
