# Supplementary material for: Reproducible high-quality perovskite single crystals by flux-regulated crystallization with a feedback loop
Source: Nat Synth. 2024 Jun 18;3(10):1212–20. doi: 10.1038/s44160-024-00576-8 (PMC11466857; doi:10.1038/s44160-024-00576-8)
Supplement: Supplementary file 1 — Supplementary Figs. 1–26, Notes 1–11 and Table 1. [file 44160_2024_576_MOESM1_ESM.pdf]

nature synthesis

Supplementary information https://doi.org/10.1038/s44160-024-00576-8

Reproducible high-quality perovskite single crystals by flux-regulated crystallization with a feedback loop

In the format provided by the authors and unedited

Table of Contents

Supplementary Note 1 – Crystallization Flux and Linear Growth Rate 2

Supplementary Note 2 – The Solubility of $MAPbBr_3$ in DMF 5

Supplementary Note 3 – Classification of Seed Crystals.. 6

Supplementary Note 4 – Details of the Flux-Regulated Crystallization System..................... 7

Supplementary Note 5 – Measurement Errors and the Validity of Data Smoothing 11

Supplementary Note 6 – Behavior of the Control Parameter During FRC 15

Supplementary Note 7 – Estimation of the Supersaturation During Crystal Growth......... 18

Supplementary Note 8 – Fitting Rocking Curve Data.. 21

Supplementary Note 9 – Two-Photon Absorption Induced Photoluminescence.................. 25

Supplementary Note 10 – Time-Resolved Photoluminescence (TRPL) Measurement 26

Supplementary Note 11 – Electrical Measurements of Crystals Grown by FRC 27

References 33

Supplementary Note 1 – Crystallization Flux and Linear Growth Rate

Crystallization flux (F [mol m^{-2} s^{-1}]) is defined as the number of molecules depositing on a unit area of the crystal surface in a unit of time. F is directly proportional to the linear growth rate (dL/dt):

Crystallization flux: F [mol m^{-2} s^{-1}]

Mass deposition flux: $F{\cdot}M_W$ [g m^{-2} s^{-1}], where M_W [g mol^{-1}] is molar weight

Linear growth rate: $F{\cdot}M_W/\rho$ [m s^{-1}] = dL/dt, where ρ [g m^{-3}] is density

Based on this unit conversion, the linear growth rate indicates the crystallization flux.

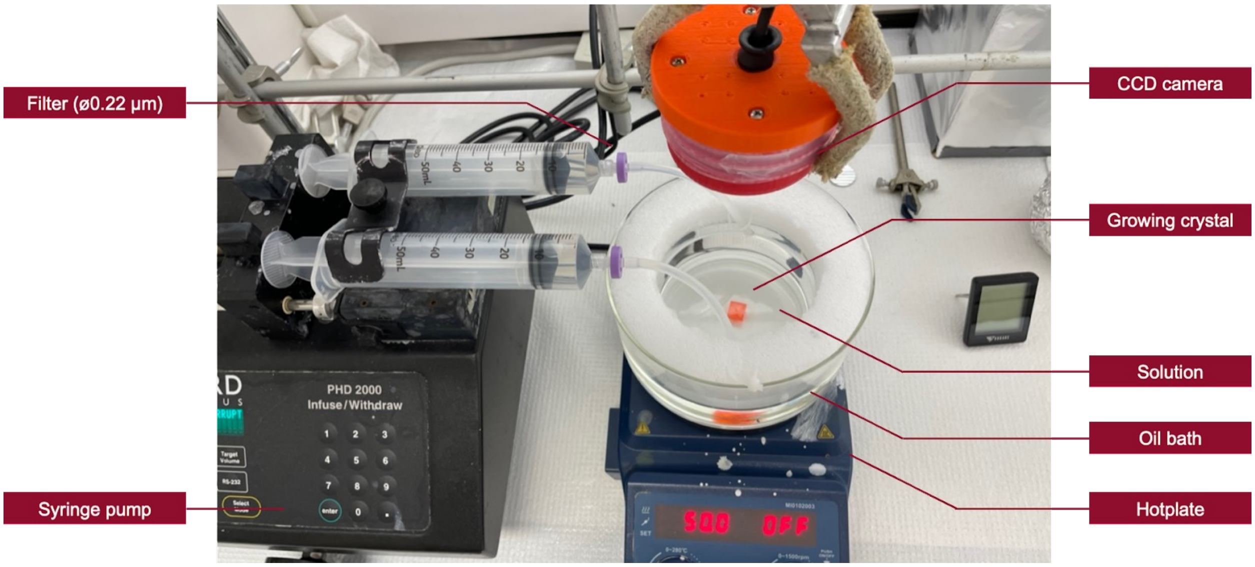

Fig. S1| A photograph of a flux-regulated crystallization system.

Camera

(Walfront) USB Camera Module 110° Wide Angle View USB Camera Module OV3660 Chip USB2.0 Output 2048x1536 15fps Support Mobile OTG High Resolution USB Camera

Syringe pump

(Harvard Apparatus) PHD 2000 Infusion/Withdraw (±0.35% accuracy)

(New Era Pump Systems) NE-4000 (±1% accuracy)

Hot plate

(Four E'S Scientific) 310°C/590°F 5 Inch Magnetic Stirrer Hot Plate

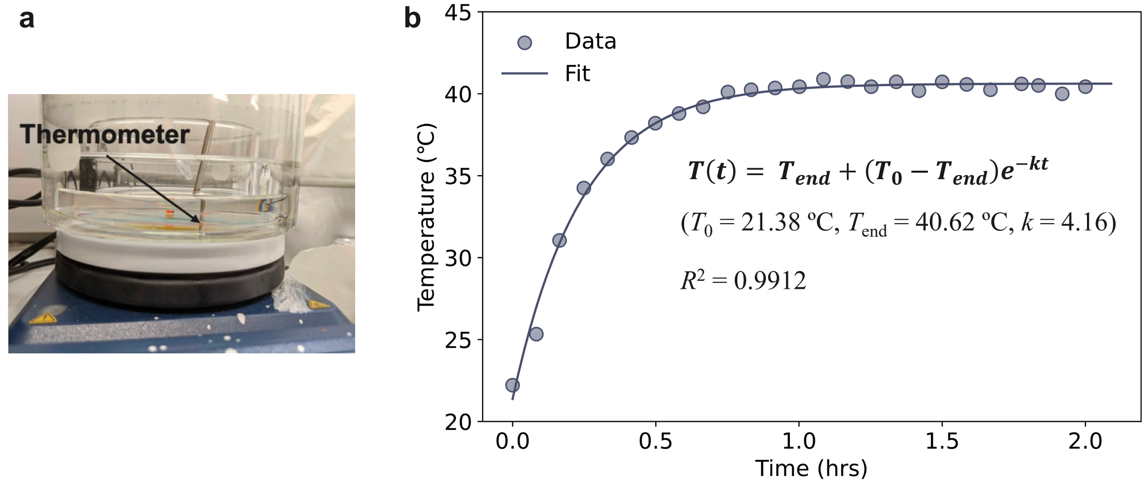

Fig. S2| The solution temperature during the crystallization process. a. A photograph of the measurement setup. A thermometer was inserted into the bottom of the solution. **b.** The solution temperature after the hotplate started heating from room temperature to displayed 50 ºC. The fitting was performed based on Newton's heating law.

Supplementary Note 2 – The Solubility of $MAPbBr_3$ in DMF

The solubility of $MAPbBr_3$ in DMF was investigated as follows: first, DMF was gradually added into a vial that had $MAPbBr_3$ powder until the powder completely dissolved at room temperature (23 ºC). Then, the vial was placed in an oil bath on a hot plate that controlled the temperature in the oil bath (**Figure S3a**). The oil bath was heated to 30 ºC and left until orange $MAPbBr_3$ precipitation was observed due to the solubility drop. We then added DMF gradually until the recrystallized $MAPbBr_3$ dissolved again. By repeating this procedure, we have obtained the solubility at 23–80 ºC range with a polynomial fitting as shown in **Figure S3b**.

The solubility at the stabilized temperature in FRC (40.62 ºC, see **Figure S2**) is estimated to be 41.37 wt.%. Therefore, the initial solution concentration was set at 41 wt.%.

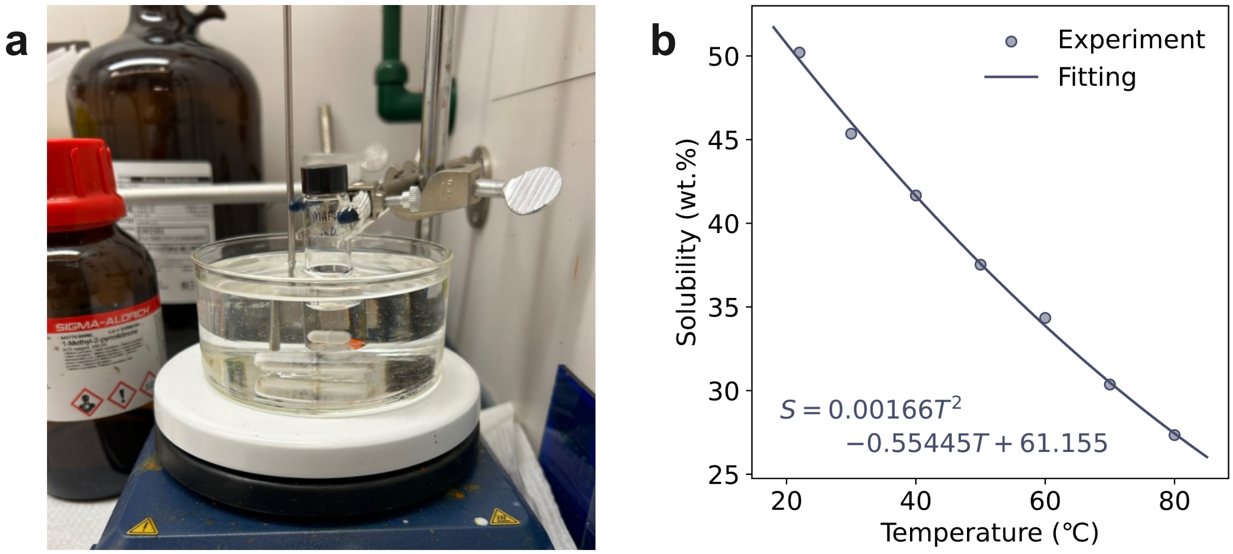

Fig. S3| a. A photograph of the solubility measurement setup. A thermometer was inserted in the oil bath. The solution and the oil were stirred to have a uniform temperature distribution **b.** The solubility curve of $MAPbBr_3$-DMF system.

Supplementary Note 3 – Classification of Seed Crystals

It is known that the quality of seed crystals affects the quality of the grown crystal.[1] To minimize the potential contribution of the quality of seed crystals to the obtained crystals, we observed the seed crystals under a polarized microscope, and then classified dark crystals to be class A (high-quality) as shown in **Figure S4**.

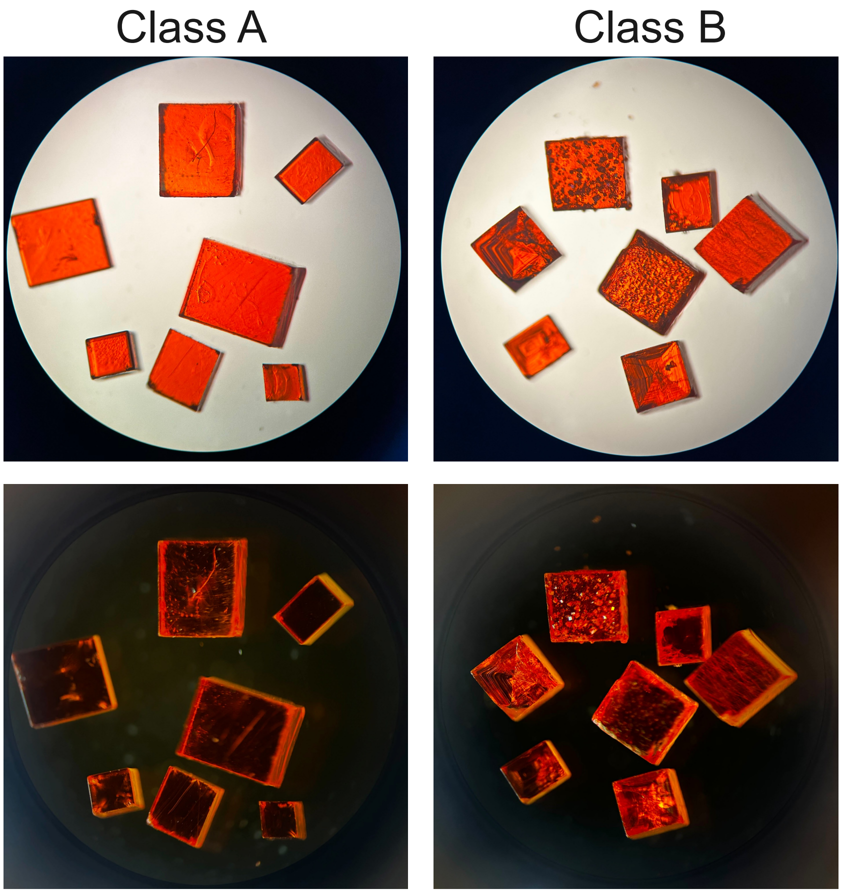

Fig. S4| Microscope images under white light (top) and polarized light (bottom) of the seed crystals that were classified to be high-quality (left: Class A, used for the crystal growth) and low-quality (right: Class B, not used for the crystal growth).

Supplementary Note 4 – Details of the Flux-Regulated Crystallization System

Main flowchart

Figure S5 shows the main flowchart of the Python code for the FRC system. After the control parameters are set, the program activates the syringe pump to infuse a solvent at a constant rate and then starts running a feedback loop consisting of (1) taking an image, (2) image processing (detection of the crystal, determination of its area, length, and others), (3) data smoothing, (4) changing the infusion rate, and (5) add the interval time for each loop. The time interval for each

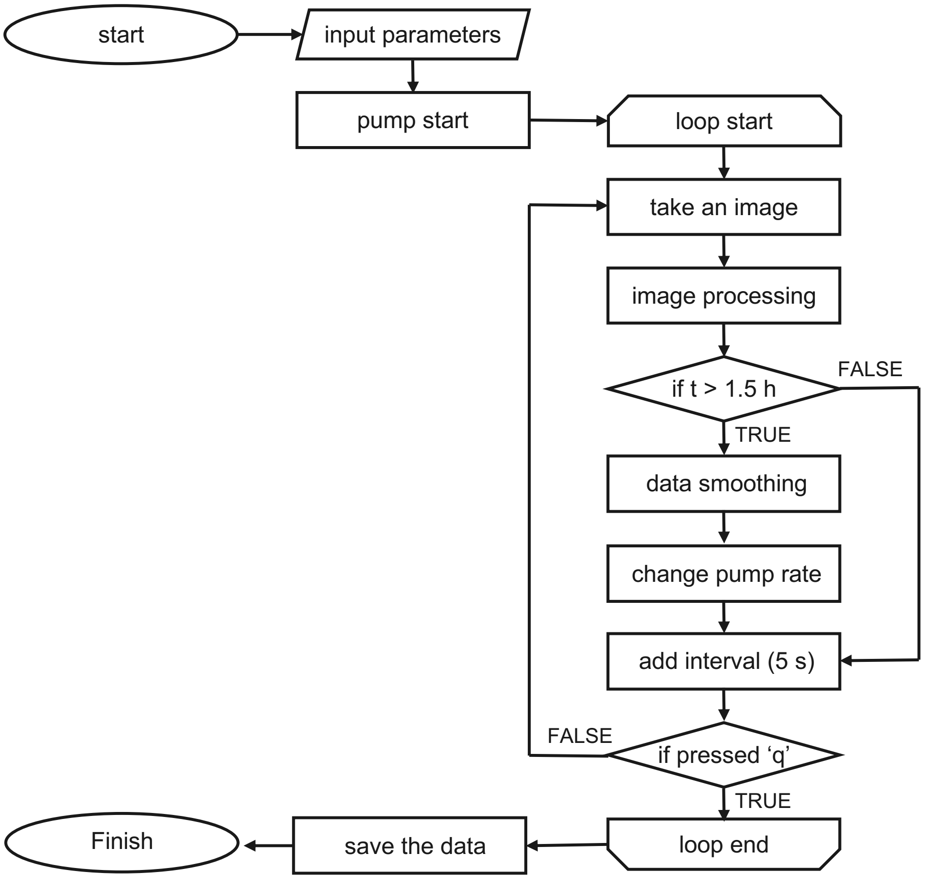

Fig. S5| The main flowchart of the Python code for the FRC system. This chart is intended to convey an overview of the system's functions; please note that it does not cover all functions. The entire code is available at https://github.com/Yuki-Haruta/2024NatureSynthesis-FRC.

loop is 5 seconds and the feedback (the change of the infusion rate) is given every 10 seconds. This feedback loop will be continued until the one manually quits the system by pressing ‘q’ on a keyboard. Note that the system is designed to infuse a solvent at a constant rate without any feedback for the first 1.5 hours because the system does not have enough data points to provide appropriate feedback due to measurement errors. Below we provide the details about each process.

Detection of the crystal

OpenCV library was used to detect the target crystal. The size of the taken image is 1920×1080 pixels. The image will be cropped in a circle shape so that the program treats only the target region, that is inside the crystallization dish. The crystal will be detected by using the cv2.findcontours method where the program detects contours based on the red-green-blue (RGB) color-values threshold. The threshold depends on the crystal color and the imaging environment so it should be optimized case by case. In our case, we used $50<R<255$, $0<G<60$, and $0<B<255$ to detect orange crystals.

Subsequent to contour detection, the area of the crystal was determined based on the count of pixels within the detected contour. A calibration procedure was undertaken using an image of a reference crystal with known area, under identical imaging conditions. This calibration yielded a conversion scale of 0.0143 mm^2/pixel. The crystal size (L) will be calculated by taking the square root of the area.

Data smoothing and calculation of the growth rate

The growth rate can be expressed as dL/dt; however, since L is obtained as discrete values, it necessitates determination through linear approximation. In this system, the slope obtained from

linear approximation of data over the past 10 minutes (120 points) was plotted as the growth rate at that time. Furthermore, to mitigate the impact of noise due to measurement errors, data on length L was smoothed using a Savitzky-Golay filter before being utilized.

How to decide the infusion rate

As shown in the manuscript, the solvent infusion rate $S_{\mathrm{inf}}(t)$ is determined by the following equation:

$$S_{\mathrm{inf}}(t) = E_{\mathrm{est}} - K_{\mathrm{P}} e(t) - K_{\mathrm{I}} \int_0^t e(\tau) d\tau - K_{\mathrm{D}} \frac{de(t)}{dt} \qquad (1)$$

where the E_{est} is the estimated evaporation rate which is a pre-determined constant value, K_{P}, K_{I}, and K_{D} are the control coefficients ([K_{P}, K_{I}, K_{D} = 3, 0.5, 2] in this work), and $e(t)$ is defined as:

$$e(t) = \mathrm{SV} - g(t) \qquad (2)$$

where the SV is a setpoint variable (a desired growth rate) and the $g(t)$ is the growth rate. The de/dt was calculated by the same method as the $g(t) = dL/dt$ calculation, that is data smoothing and the linear approximation. The $\int_0^t e(\tau) d\tau$ was calculated by using the trapezoidal approximation as indicated by the following equation:

$$\int_0^t e(\tau) d\tau \approx \frac{\Delta t}{2} \sum_{i=1}^{n} \{e(t_{i-1}) + e(t_i)\} \qquad (3)$$

The volume of the infused solvent was recorded by adding a product of interval time (s) and infusion rate (mL/h).

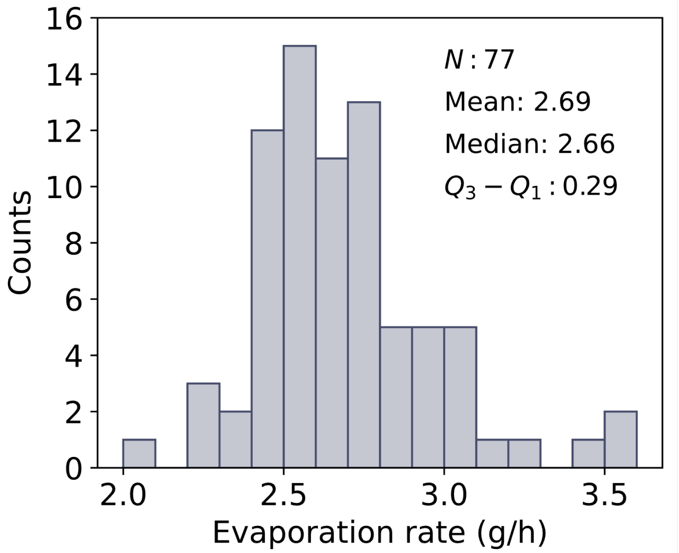

Fig. S6| The histogram of solvent evaporation rates in 77 experiments.

Supplementary Note 5 – Measurement Errors and the Validity of Data Smoothing

To evaluate the measurement error in image processing, we placed crystals with known sizes (0.85, 1.79, 6.25, and 7.40 mm) on a hotplate, then captured 71 images for each crystal and derived the length using the same program employed for the crystal growth. As these crystals are not in a solution, their size should not change. As shown in **Figure S7**, the measured values fluctuated with the standard deviation of 0.04 mm for the small crystal (0.85 mm) and 0.01 mm for bigger ones (>1.79 mm). Thus, we concluded that the image processing has nearly 0.01–0.04 mm of measurement errors depending on the crystal size.

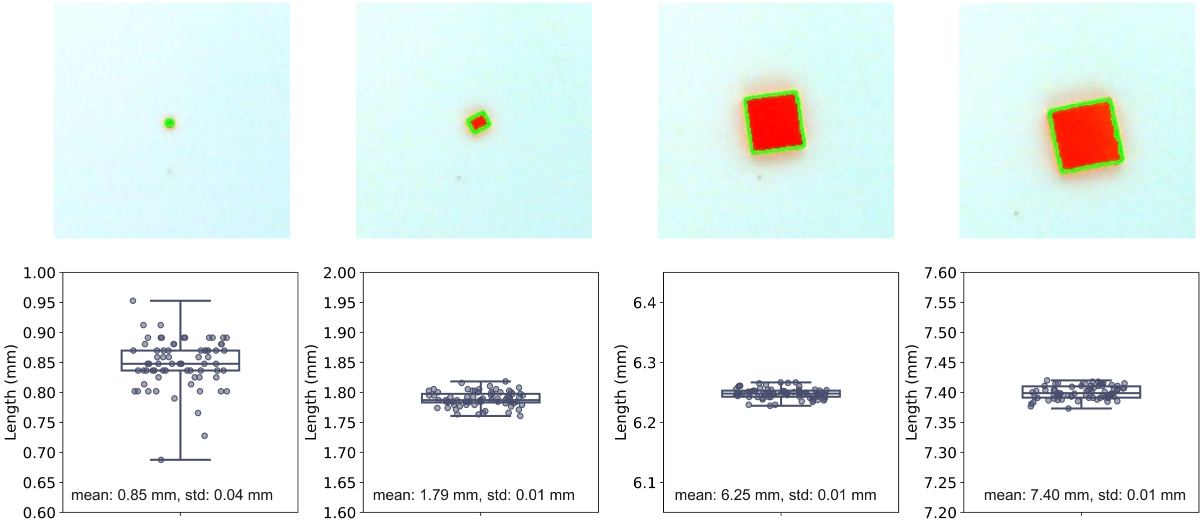

Fig. S7| Representative captured images of crystals placed on a hotplate and the box plots of their measured length from 71 images. (center line, median; box limits, upper and lower quartiles; whiskers, minimum and maximum values; points, source data)

To prevent the influence of measurement noise in image processing on the feedback control, it should be removed by data smoothing. **Figure S8a** shows the measured length and the smoothed length in the SV0.2-FRC (the same experiment as in **Figures 2b** and **2c)**. The smoothed data appears to trace the original data points well.

To examine whether too much information has been lost by data smoothing, the errors between the measured length and the smoothed length were analyzed as shown in **Figure S8b**. The errors slightly decreased as the crystal size increased, in agreement with the trend observed in the previous analysis (**Figure S7**). As shown in the histogram of the errors (**Figure S8c**), the errors follow a Gaussian distribution (mean: 0.000, stdev: 0.010 mm), which also agrees well with the previous analysis (**Figure S7**). Therefore, we concluded that the data smoothing only eliminated the measurement errors in image processing.

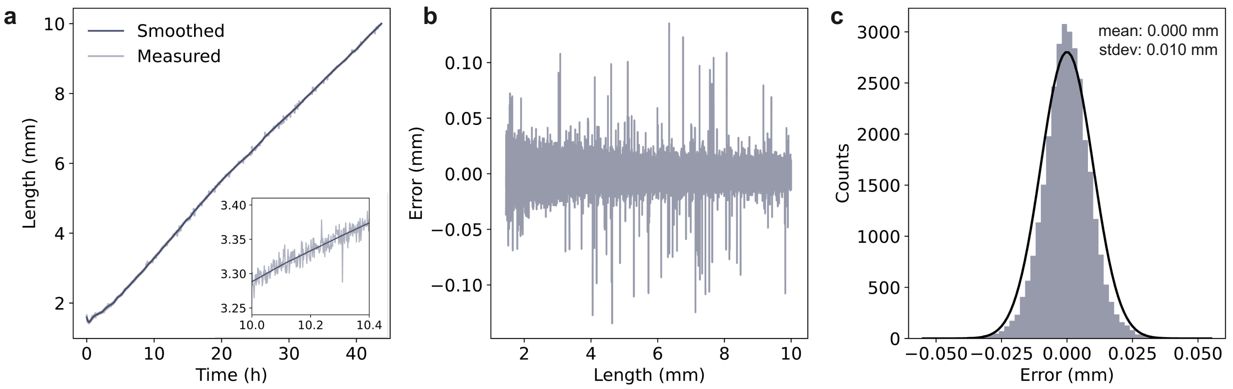

Fig. S8| a. The measured length and smoothed length in the SV0.2-FRC experiment. The inset shows the selected data. **b.** The measurement errors (the measured length – the smoothed length) against the smoothed length. **c.** The histogram of the measurement errors. The line shows the Gaussian fittings with the mean of 0.000 mm and the standard deviation of 0.010 mm.

Figure S9 shows the measured growth rate and the smoothed growth rate in the SV0.2-FRC. The growth rate was determined as a slope of the linear regression applied to the preceding 10 minutes of $L(t)$ data (**Supplementary Note 4**). The data smoothing for $L(t)$ effectively removed the measurement errors as discussed above, yet the short interval (10 minutes) could still lead to the noise in the growth rate calculation. Therefore, the FRC system also smoothens the growth rate data. The smoothed data agreed well with the growth rate calculated for the longer interval (1 hour) based on the smoothed L(t) data, confirming that the growth rate obtained by smoothing the data is a good reflection of the actual growth rate.

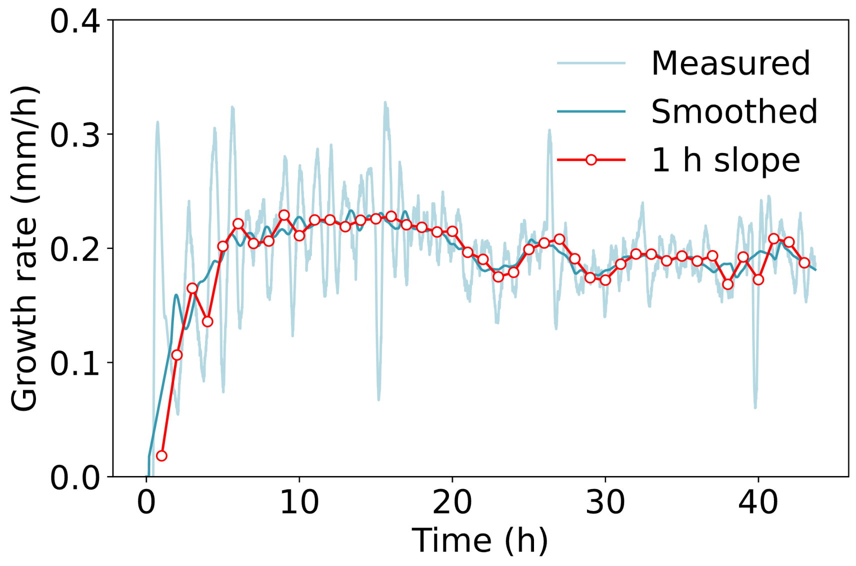

Fig. S9| The measured and smoothed growth rate in the SV0.2-FRC along with post-calculated growth rate which is derived from the smoothed $L(t)$ data but using 1 hour interval instead of 10 minutes.

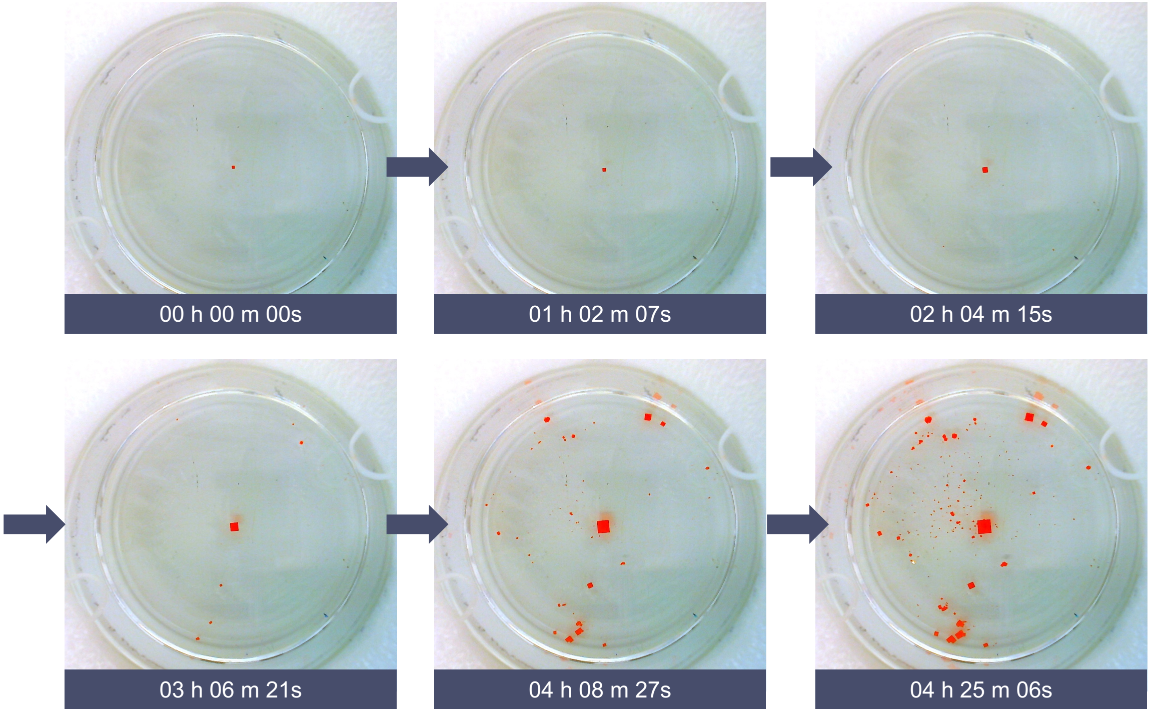

Fig. S10| Photographs of the crystallization dish without feedback control and solvent infusion, showing many nucleation after 4 hours.

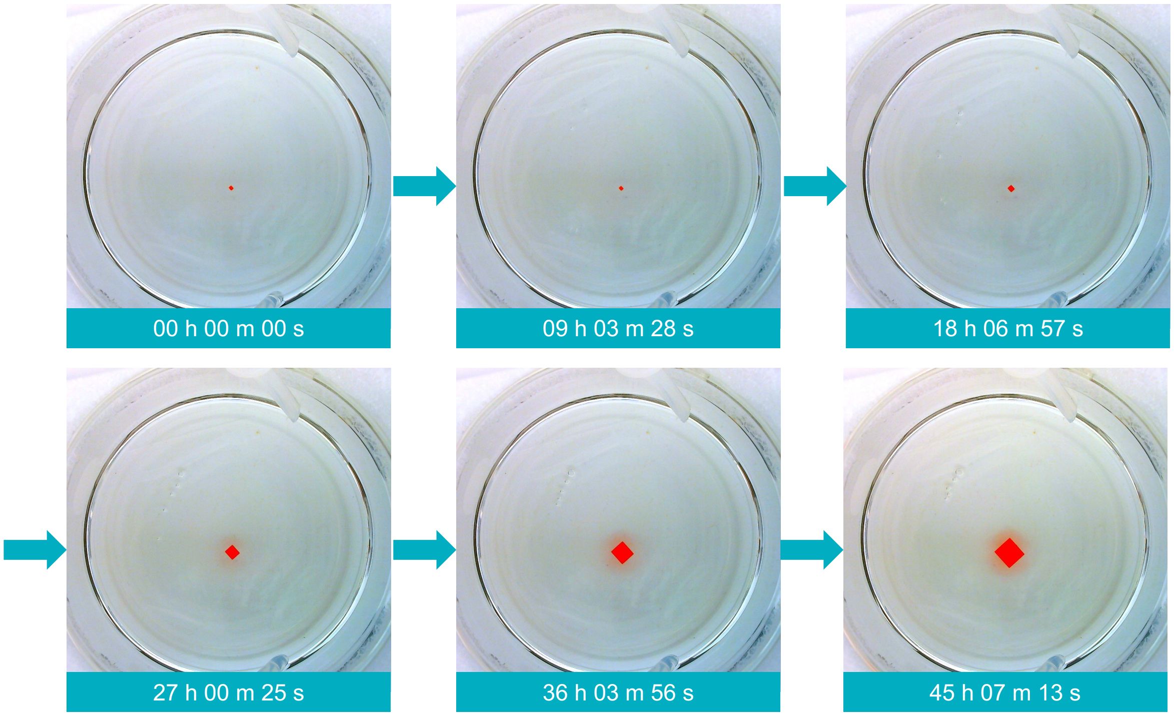

Fig. S11| Photographs of the crystallization dish with the feedback control (SV = 0.2 mm h^{-1}).

Supplementary Note 6 – Behavior of the Control Parameter During FRC

To understand how the FRC system controls the growth rate, we plotted the linear growth rate ($dL(t)/dt$) and infusion rate ($S_{\mathrm{inf}}(t)$), specifically focusing on the time interval before the growth rate was stabilized (**Figure S12**). The dashed lines represent $dL(t)/dt$ = SV and $S_{\mathrm{inf}}(t) = E_{\mathrm{act}}$ (the actual evaporation rate, 2.712 mL/h). At point A_1 (t = 2.45 h), the infusion rate surpassed the evaporation rate, signifying a decline in average solution concentration from this juncture. Nonetheless, this input from the FRC system resulted in the start of a decrease of the $dL(t)/dt$ at point A_3 (t = 3.10 h) which is delayed by 39 minutes. Conversely, the $e(t)$ started increasing at point B_3 (t = 4.04 h) which was ~48 minutes after the solvent infusion rate got below the evaporation rate at point B_1 (t = 3.24 h). These control delays could be attributed to the slow diffusion in the solution, that is, the infused solvent takes 23−48 mins (time differences between [A_1, B_1 ,…E_1] and [A_3, B_3 ,…E_3]) to effect on the solution concentration near the target crystal.

Noteworthy is that the infusion rate peaks anticipate the control error peaks by 9 to 16 minutes, a proactive correction afforded by the D-term in the PID equation. Absent the D-term, the infusion rate curve would largely mimic the control error curve, since the control equation is predominantly P-controlled. Observing the interval between A_1 and A_3, the peak infusion rate at A_2 precedes the $dL(t)/dt$ peak at A_3 by 16 minutes. This preemptive action mitigates excessive overshoot in the control error, thereby indicating a more expedient rectification of the system error.

We note that the PID coefficients ([K_P, K_I, K_D] = [3, 0.5, 2]) were manually optimized based on some trials. Hence, the following equation was used to determine the infusion rate in this experiment:

$$S_{\text{inf}}(t) = 2.62 - 3e(t) - 0.5\int_0^t e(\tau)d\tau - 2\frac{de(t)}{dt}$$

Estimated evaporation rate was 2.62 ml/h. There can be still room to further optimize PID coefficients.

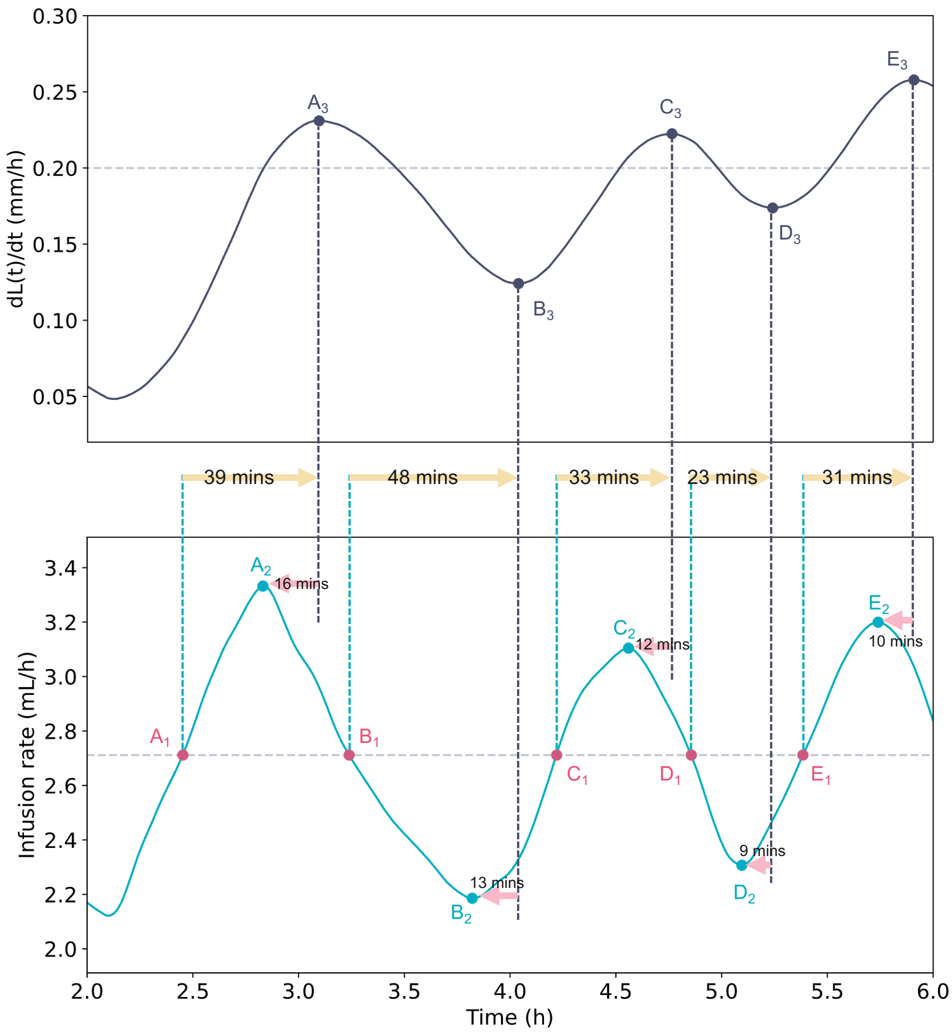

Fig. S12| The $dL(t)/dt$ and $S_{\text{inf}}(t)$ during the FRC. The dashed lines in top and bottom figures represent $dL(t)/dt = 0.2$ and $S_{\text{inf}}(t) = E_{\text{act}}$, respectively. Red ($A_1$–$E_1$), blue ($A_2$–$E_2$), and dark blue ($A_3$–$E_3$) points represent the points when the $S_{\text{inf}}(t) = E_{\text{act}}$, peaks for $S_{\text{inf}}(t)$ and $dL(t)/dt$, respectively.

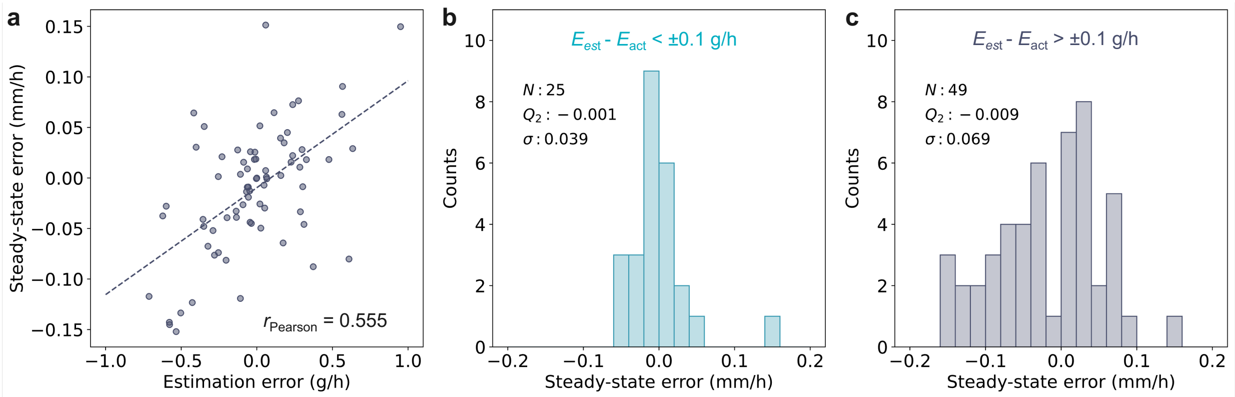

Fig. S13| a. Correlation between the steady-state errors (SV – sPV) and the evaporation rate estimation error ($E_{est} – E_{act}$). **b–c.** The histograms of the steady-state errors (SV – sPV) when the evaporation estimation error ($E_{est} – E_{act}$) is below (b) and above (c) 0.1 g h^{-1}.

Supplementary Note 7 – Estimation of the Supersaturation During Crystal Growth

The mass concentration during crystal growth ($C(t)$) can be expressed as follows:

$$C(t) = \frac{m_{solute}(0) - m_{deposition}(t)}{m_{solution}(0) - m_{evaporation}(t) + m_{infusion}(t) - m_{deposition}(t)}$$

where m_{solute}, m_{solution}, $m_{\text{deposition}}$, $m_{\text{evaporation}}$, and m_{infusion} are the mass of solute, solution, deposited crystals, evaporated solvent, and infused solvent, respectively. While m_{infusion} can be recorded by the programmable pump, the $m_{\text{evaporation}}$ and the $m_{\text{deposition}}$ must be estimated. We estimated the $m_{\text{evaporation}}$ by presuming the evaporation rate was constant during crystal growth. Thus, it is a product of the evaporation rate and the time. For the $m_{\text{deposition}}$, the total mass (m_{total}) of the crystal in the solution was first estimated by using the following equation:

$$m_{total}(t) = \rho \sum_{i}^{n} V_i(t) = \rho \sum_{i=0}^{n} a(A_i(t))^{\frac{3}{2}}$$

where ρ is the density of $MAPbBr_3$ (3.8 g cm^{-3}), n is the number of crystals detected by the program, $V_{\text{i}}(t)$ is the volume of each crystal, a is the correction factor which will be calibrated later, and $A_{\text{i}}(t)$ is the area of each crystal. Then, the mass of deposition ($m_{\text{deposition}}$) is calculated as the difference between $m_{\text{total}}(t)$ and the mass of the seed crystal ($m_{\text{total}}(0)$) as follows.

$$m_{deposition}(t) = m_{total}(t) - m_{total}(0)$$

Now, the solution concentration can be estimated by substituting the variables to the first equation.

On the other hand, we do not need estimation for the final solution concentration (C_{final}) because it can be calculated by measuring the total mass of deposited crystals, and changes in the mass of

the syringes and the container including the solution before and after the crystal growth. Therefore, we modified the correction factor (a) so that the calculated $C(t_{\text{final}})$ would be equal to the measured C_{final}.

Additionally, combining the fitting equation for the temperature profile ($T(t)$, **Figure S2**) and the solubility curve ($S(T)$, **Figure S3**), we can calculate the solubility as a function of the time ($S(t)$), and thus, supersaturation level ($\sigma(t)$) over time can be calculated as follows:

$$\sigma(t) = \frac{C(t) - S(t)}{S(t)}$$

According to the above-discussed calculation, **Figure S14** shows the estimated supersaturation level vs. the crystal linear growth rate in the experiments corresponding to the data in **Figure 2a**.

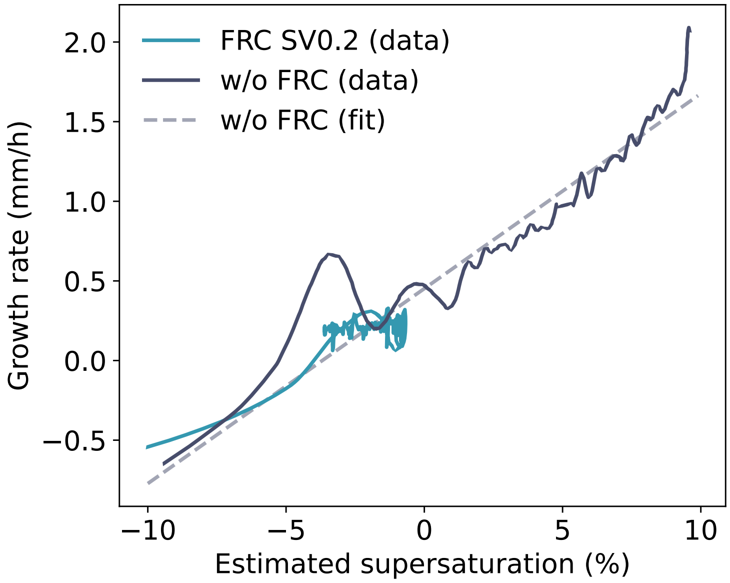

Fig. S14| Estimated supersaturation vs. growth rate in the crystal growth with FRC (SV0.2) and without FRC.

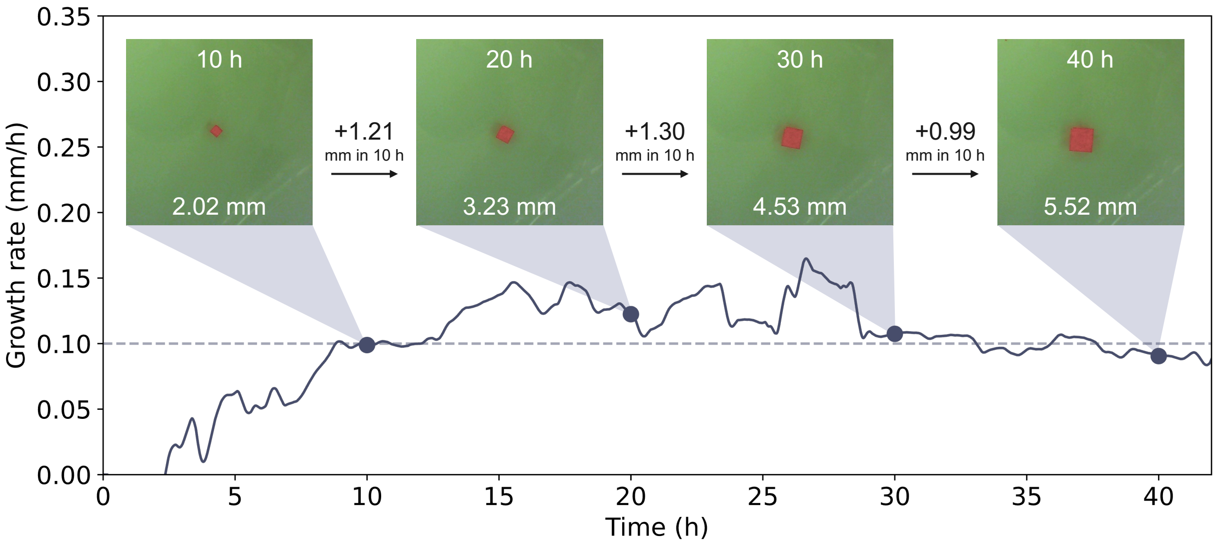

Fig. S15| The growth rate of $CsPbBr_3$ by the FRC system. The insets show the images of the growing crystal and the size at 10, 20, 30, and 40 hours which gives average growth rates of 0.12, 0.13, and 0.10 mm h^{-1} for each 10-hour interval.

Supplementary Note 8 – Fitting Rocking Curve Data

The experimental data were fitted by using PearsonVII function which is defined as follows:

$$I(\omega) = I_0 + A\frac{2\Gamma(\mu)\sqrt{2^{-\mu}-1}}{\sqrt{\pi}\Gamma(\mu-0.5)w}\left[1+4\frac{2^{-\mu}-1}{w^2}(\omega-\omega_0)^2\right]^{-\mu}$$

where $\Gamma(x)$ is the gamma function, I_0 is the peak intensity, ω_0 is the peak position, A is the peak area, w is the peak width (FWHM), and μ is the profile shape factor.

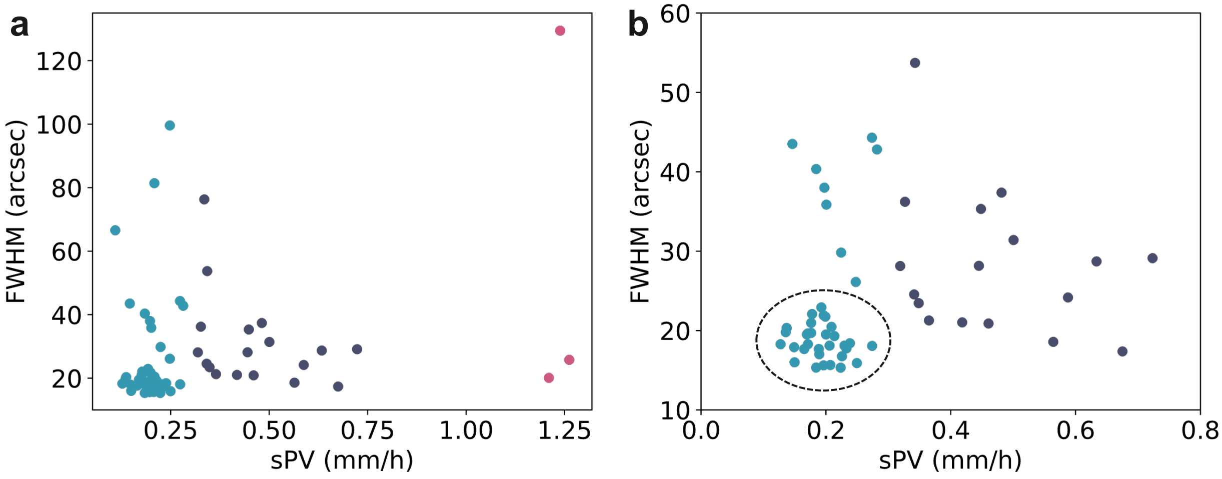

Fig. S16| a. FWHM of (100)-rocking curves of the crystals by FRC method (blue: sPV<0.3, dark blue: sPV>0.3) and solvent evaporation method (red). **b.** A cropped figure of panel **a**. The dashed circle indicates a cluster of high-crystallinity crystals grown at slow growth rates.

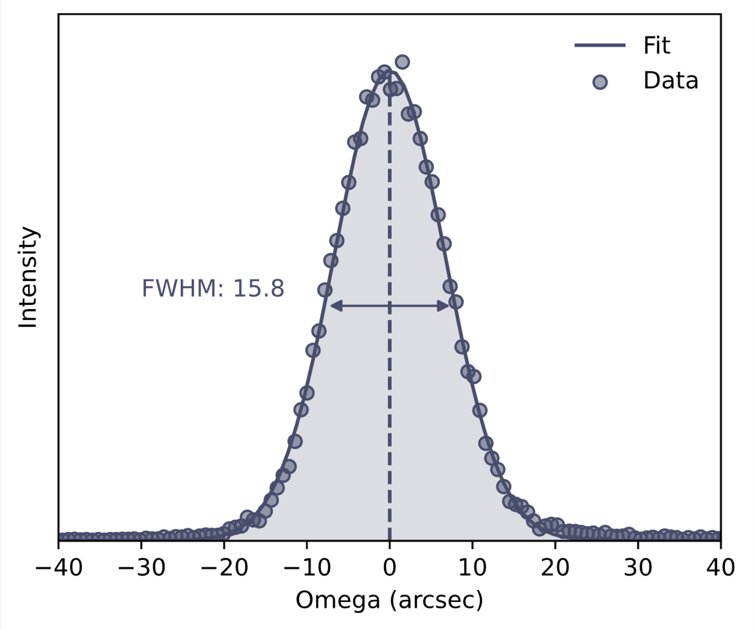

Fig. S17| The rocking curve of a (400) plane of a commercial Si-wafer (ALPHA Nanotech, 5 mm × 5 mm Ultra-Flat Single-Sided Diced Silicon Chip, Grade: Prime/CZ Virgin, Type/Dopant: P/Boron, Orientation: <100>).

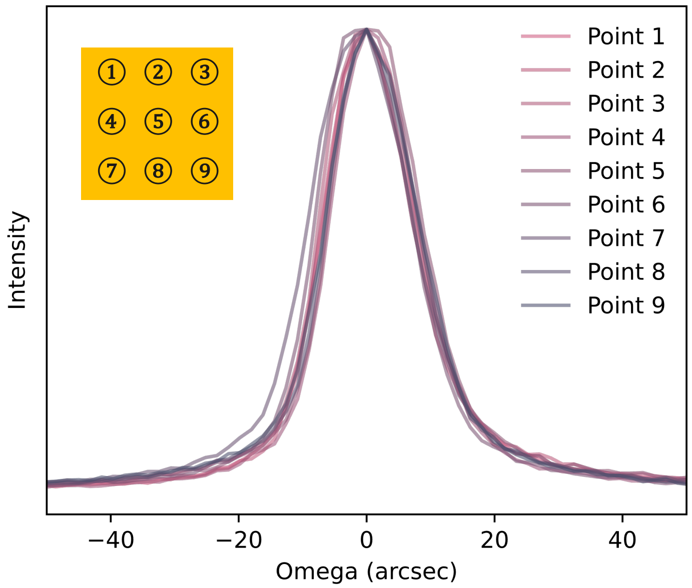

Fig. S18| (100) rocking curves at 9 different points.

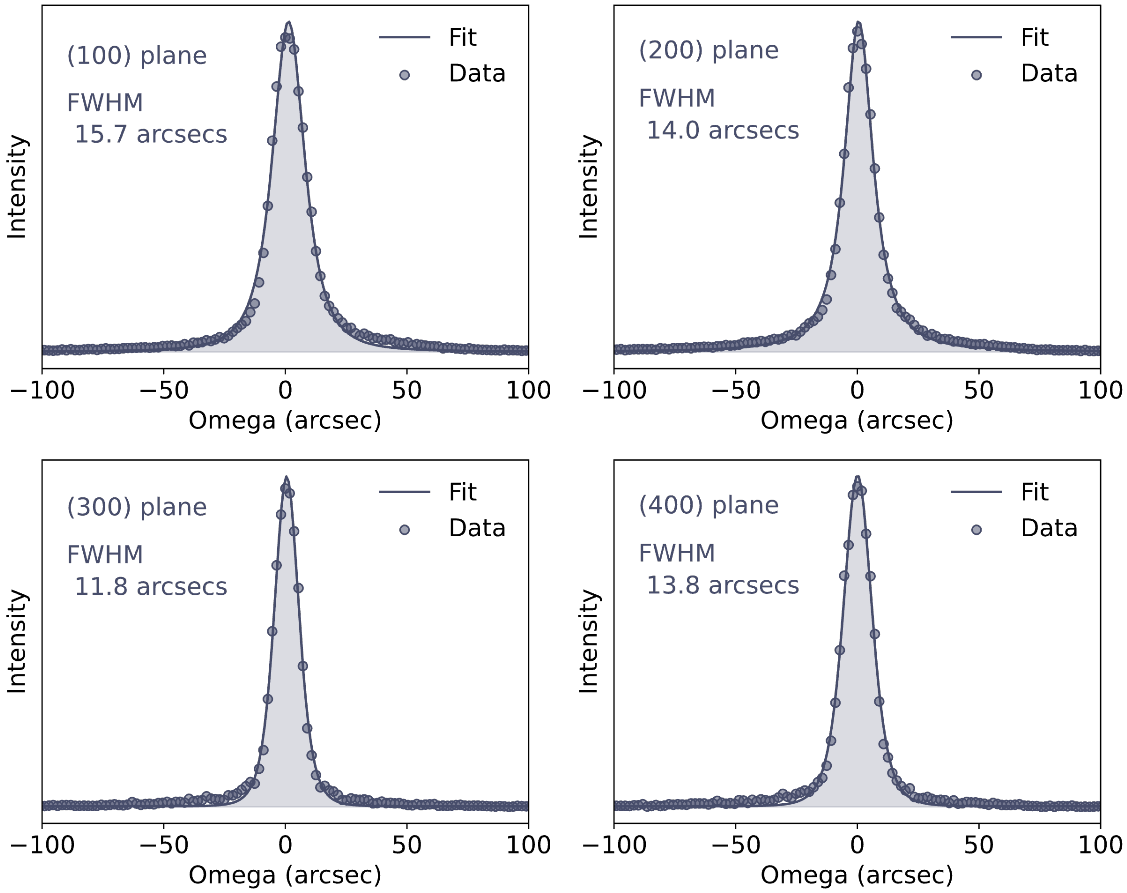

Fig. S19| Rocking curves for (100), (200), (300), and (400) planes.

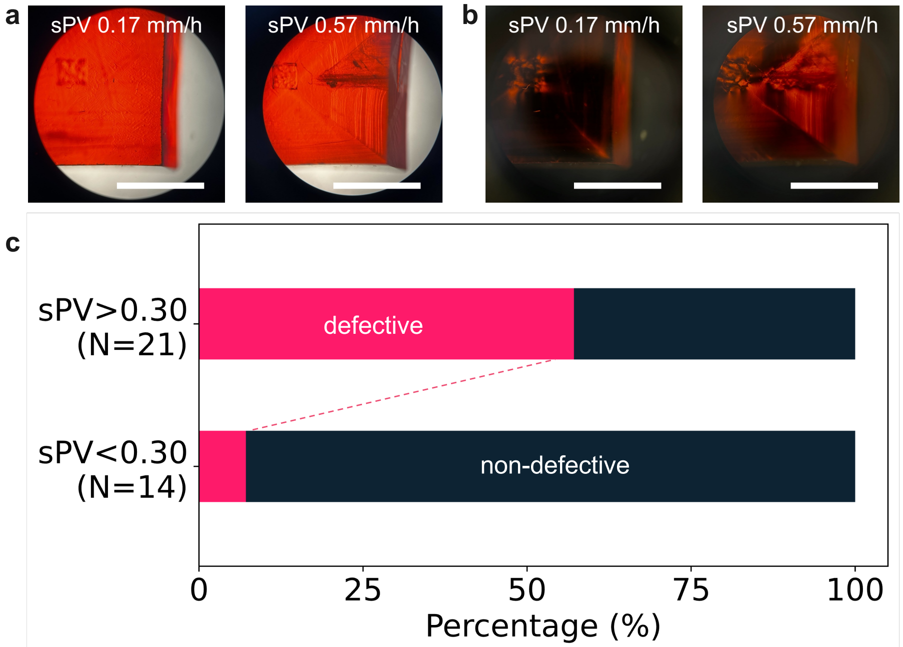

Fig. S20| a–b. Microscope images of sPV-0.17 mm h^{-1} and sPV-0.57 mm h^{-1} crystals under (a) white light and (b) polarized light. The scale bar is 2 mm. **c.** Percentage of the defective crystals obtained at slow (sPV < 0.30 mm h^{-1}) and fast (sPV > 0.30 mm h^{-1}) growth rates.

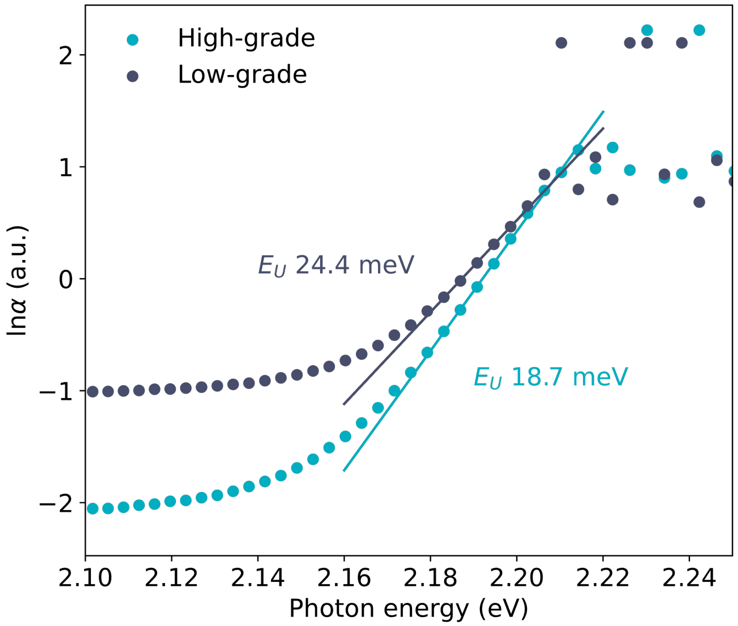

Fig. S21| Urbach energy extraction for high-grade and low-grade single crystals. The values when the photon energy is over 2.21 eV is noisy due to the detection limit of the instrument.

Supplementary Note 9 – Two-Photon Absorption Induced Photoluminescence

The observed red-shifted and asymmetric peaks in the two-photon absorption induced photoluminescence (TPA-PL) of the crystals, in comparison to the single-photon absorption induced photoluminescence (SPA-PL), can be attributed to the phenomena of re-absorption.[2] This is evident from the transmittance spectra of the crystal, which suggests that a portion of the photoluminescence emitted from the crystal's bulk is re-absorbed by the crystal material as it exits. This re-absorption process significantly influences the spectral characteristics of the TPA-PL, resulting in the observed alterations in peak symmetry and position.

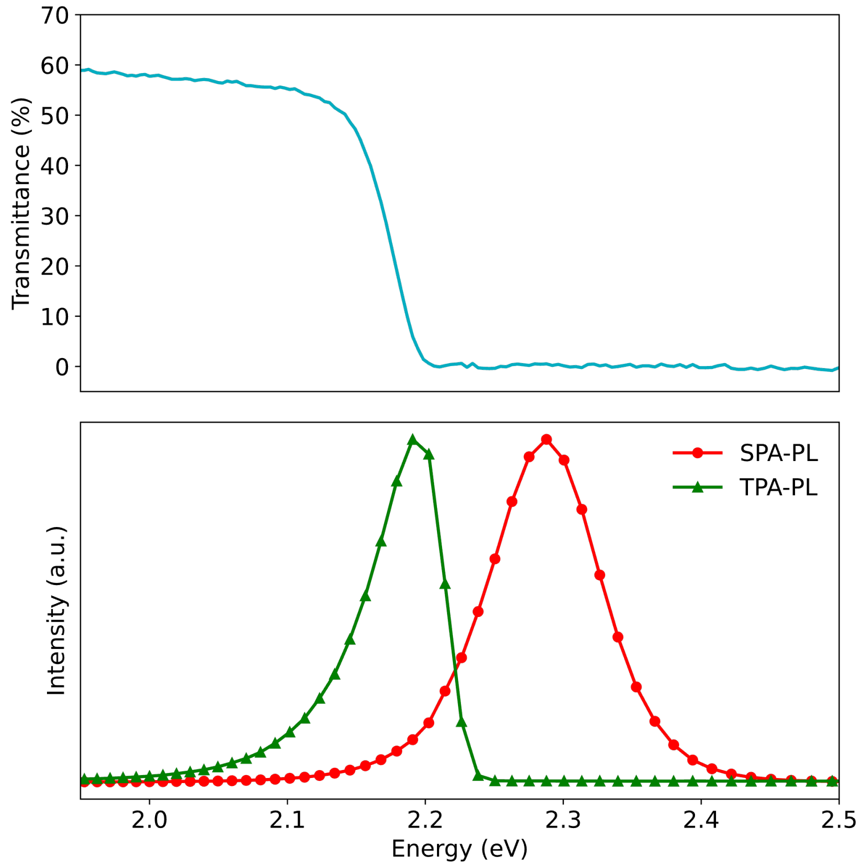

Fig. S22| Transmittance, SPA-PL, and TPA-PL spectra of the high-grade crystal corresponding to Figure 5 d–f in the manuscript.

Supplementary Note 10 – Time-Resolved Photoluminescence (TRPL) Measurement

TRPL measurements were performed by a single photon counting system (Edinburgh Instruments OB920). The samples were excited using a 510 nm pico-second pulsed laser. PL emission was collected at 540 nm using a 16 nm bandwidth monochromator and 515 nm long-pass filter. To eliminate the effects of any surface residues, the samples were cleaved, and the cleaved surfaces were measured. As shown in **Figure S23a**, we have measured two high-grade and two low-grade crystals [HG-1: sPV 0.15 mm s^{-1}, FWHM 16.0 arcsecs], [HG-2: sPV 0.17 mm s^{-1}, FWHM 18.3 arcsecs], [LG-1: sPV 0.33 mm s^{-1}, FWHM 36.2 arcsecs] and [LG-2: sPV 0.36 mm s^{-1}, FWHM 21.3 arcsecs]. The TRPL decay curves were well-fitted by tri-exponential decay. The three obtained decay kinetics are attributed to radiative recombination occurring on the surface, surface-bulk transition layer, and bulk in the shortest order;[3] so the longest decay time was extracted as a bulk component. For the statistical analysis, TRPL decay was measured at three different locations for each sample (**Figure S23b**). The average bulk lifetimes were 409, 281, 191, and 160 ns for HG-1, HG-2, LG-1, and LG-2, respectively.

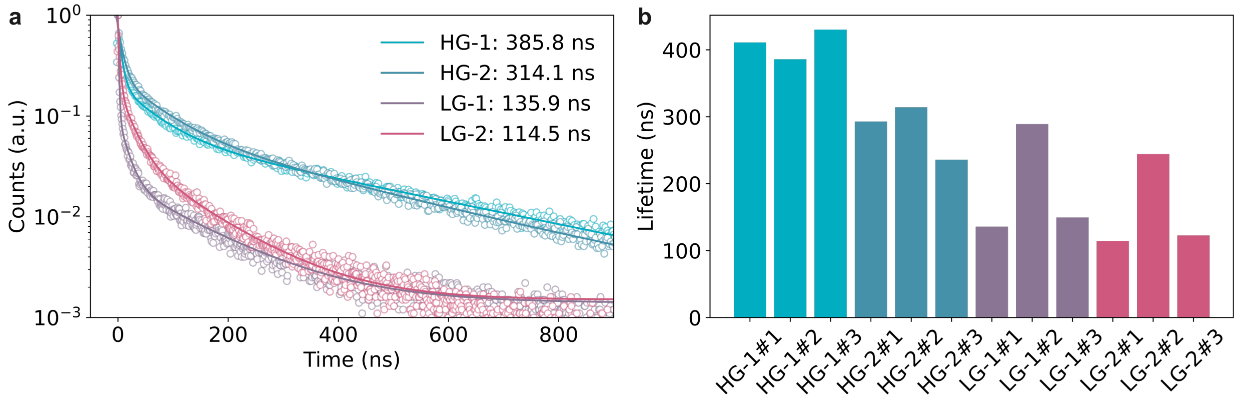

Fig. S23| a. Time-resolved PL decay curves for two high-grade crystals (HG-1, HG-2) and two low-grade crystals (LG-1, LG-2). The solid lines show the fitting results with a tri-exponential decay function. **b.** Bulk carrier lifetime of each sample measured at different locations.

Supplementary Note 11 – Electrical Measurements of Crystals Grown by FRC

Device fabrication for electrical measurements

For electrical measurements, as-grown $MAPbBr_3$ crystals were polished by polishing papers and gold electrodes were deposited by thermal evaporation to fabricate Au/$MAPbBr_3$/Au sandwiched devices that have 80 nm gold on one side and semi-transparent 40 nm gold on the other side.

Time-of-flight measurements

To evaluate the mobility of the crystals, the Time-of-Flight (ToF) measurements were conducted with a nano-second laser (wavelength; 500 nm) while applying different voltages of 10–100 V to the device, characterizing the transport of photogenerated charge carriers across the bulk of the crystal. The light was irradiated from the semitransparent Au electrode while applying a positive bias on this electrode and a negative bias on the other electrode so hole transportation would be observed.

Figure S24a presents a graph of the transient current measured across different biases. We define the transit time, τ, as the point where a notable change occurs in the transient currents, which we have highlighted with black circles for clarity. In the inset of the figure, we plot these transit time values as a function of the inverse voltage (V^{-1}), providing a clear visualization of their relationship. The mobility (μ) of the carriers within the material is calculated directly from the transit time, the thickness of the crystal (d), and the applied voltage (V), using the formula $\mu = d^2/(V\tau)$. By performing a linear regression analysis of the transit time (τ) against the inverse voltage (V^{-1}), we derived an estimated mobility value of 89.5±25 cm^2 V^{-1} s^{-1}.

We then conducted ToF measurements for different samples, varying in sPV values, as shown in **Figure S24b**. The slow-grown crystals (sPV<0.3) showed a higher average mobility of 97.4 cm^2 V^{-1} s^{-1} (or 73.2 cm^2 V^{-1} s^{-1} in case the exceptional point [217 cm^2 V^{-1} s^{-1}] is removed from the calculation) compared to fast-grown crystals (avg: 52.6 cm^2 V^{-1} s^{-1}).

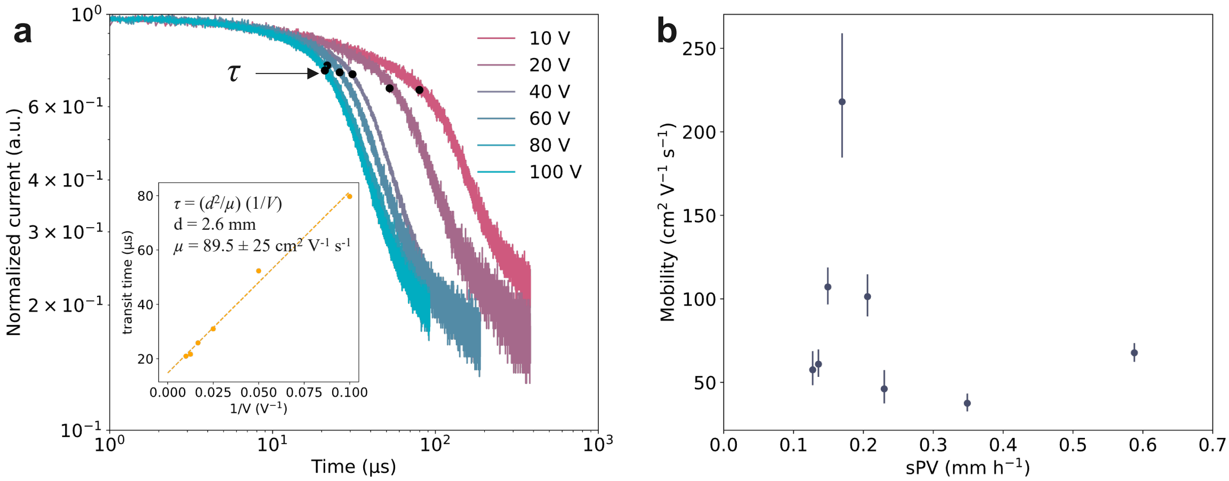

Fig. S24| a. The transient current after photoexcitation at different biases (10–100 V). The transit time τ is identified by the corner in each trace and marked by the black circle. The inset shows a linear fit of transit time versus inverse voltage. **b.** Hole mobilities measured by ToF vs. the crystal's stabilized growth rate (sPV). The error bars represent the errors in calculated mobility which was evaluated by considering the standard deviation of the slope in the linear regression (the inset of panel a) and the errors in thickness measurement.

μτ-products evaluation by Hecht fitting

The mobility-lifetime product ($\mu\tau$) is derived from the Hecht equation that describes the charge collection efficiency (CCE) of photogenerated carriers at different biases as follows:

$$\mathrm{CCE}(V) = \frac{Q(V)}{Q_0} = \frac{\mu\tau V}{d^2}\left(1 - \exp\left(-\frac{d^2}{\mu\tau V}\right)\right)$$

where $Q(V)$ is collected charges at a bias of V, Q_0 is the photogenerated carriers, and d is the sample thickness. The carriers were generated from the semitransparent electrode side by illuminating

1SUN visible light for 15 seconds using a solar simulator. **Figure S25** shows the results of Hecht fitting with the samples' sPV and FWHM values. The best $\mu\tau$ of 1.77 × 10^{-2} cm^2 V^{-1} was obtained from the slow-grown crystal (sPV: 0.24 mm h^{-1}, FWHM: 18.4 arcsec). We note that these values are higher than the $\mu\tau$ products expected from the ToF and TRPL measurements (10^2 cm^2 V^{-1} s^{-1} in ToF and 10^3 ns in TRPL derives 10^{-4} cm^2 V^{-1} of $\mu\tau$ products); this is because the carrier transport was likely unaffected by trap states of the crystal as they would have been filled by photogenerated carriers under the steady-state illumination.

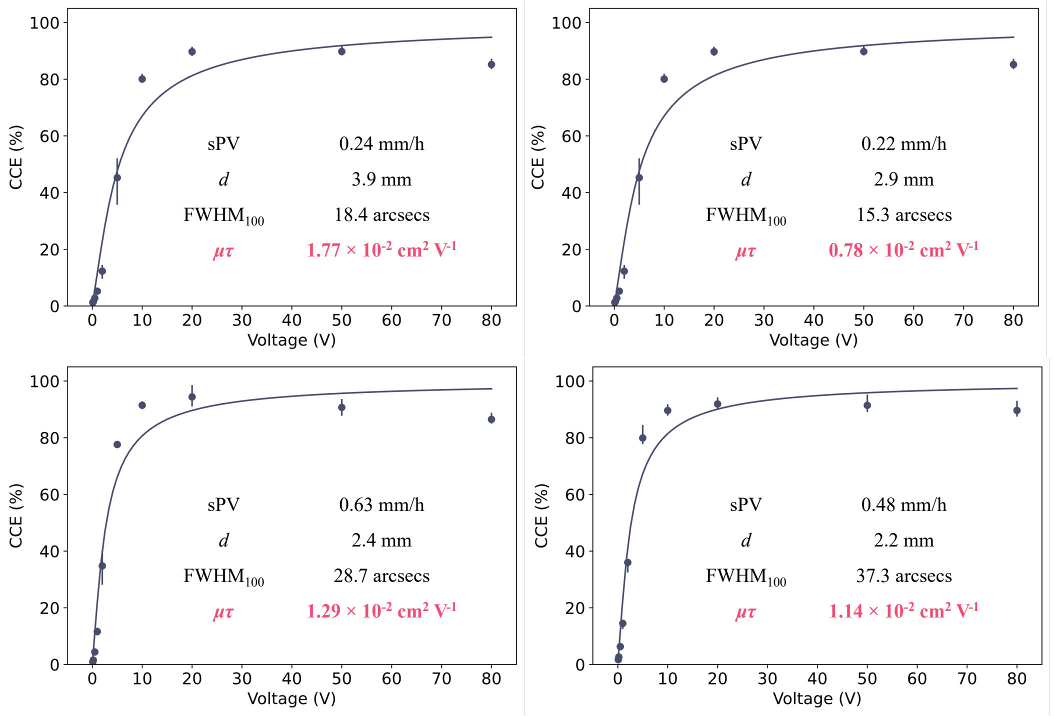

Fig. S25| Charge collection efficiency (CCE) of photogenerated carriers in four different samples. The dots show the CCE, and the lines show the fitting result based on the Hecht equation. The error bars represent the fluctuation of the photocurrent during each illumination.

Device fabrication for X-ray detectors

The X-ray detectors with a device configuration of Au/$MAPbBr_3$/Ga were fabricated with sPV-0.17 and sPV-0.48 mm/h crystals. The thicknesses were 2.4 mm and 3.1 mm, respectively. The Au electrodes (80 nm thick) were thermally evaporated on the surface of the polished $MAPbBr_3$ crystals. Then, Ga electrodes were deposited on the opposite surface by using liquified Ga and solidifying it at room temperature. The electrode area was set to 0.044 cm^2.

X-ray detection performance

For the X-ray measurement, a Comet MXR-160/22 X-ray tube[4] with a tungsten anode was used as an X-ray source. The X-ray tube voltage was set to 40 kV. The dose rate was controlled by using a 0.36 mm thick Cu plate as an attenuator and changing the tube current. The dose rate was calibrated by an ion chamber dosimeter. A Keithley 2450 sourcemeter was used for applying the bias and measuring the current.

Figure S26 shows the results of X-ray detection measurements. As shown in **Figures S26a** and **S26b**, the sPV-0.17 device showed relatively lower responses to the X-rays with the dose rates of 4.8–33.6 μGy s^{-1} compared to the sPV-0.48 device. The sensitivities at 50 V mm^{-1} (a slope of the linear fitting) were 1,397 and 1,603 μC Gy^{-1} cm^{-2} for sPV-0.17 and sPV-0.48 devices, respectively (**Figure S26c**). This is probably because of the difference in their thicknesses (2.4 mm vs. 3.1 mm). Although the sPV-0.48 device showed higher sensitivities than sPV-0.17 device, it showed poor R-square values with 0.917–0.982 while sPV-0.17 showed 0.952–0.999 (**Figure S26d**), indicating the sPV-0.17 device showed a better linear response, thus is a better X-ray detector.

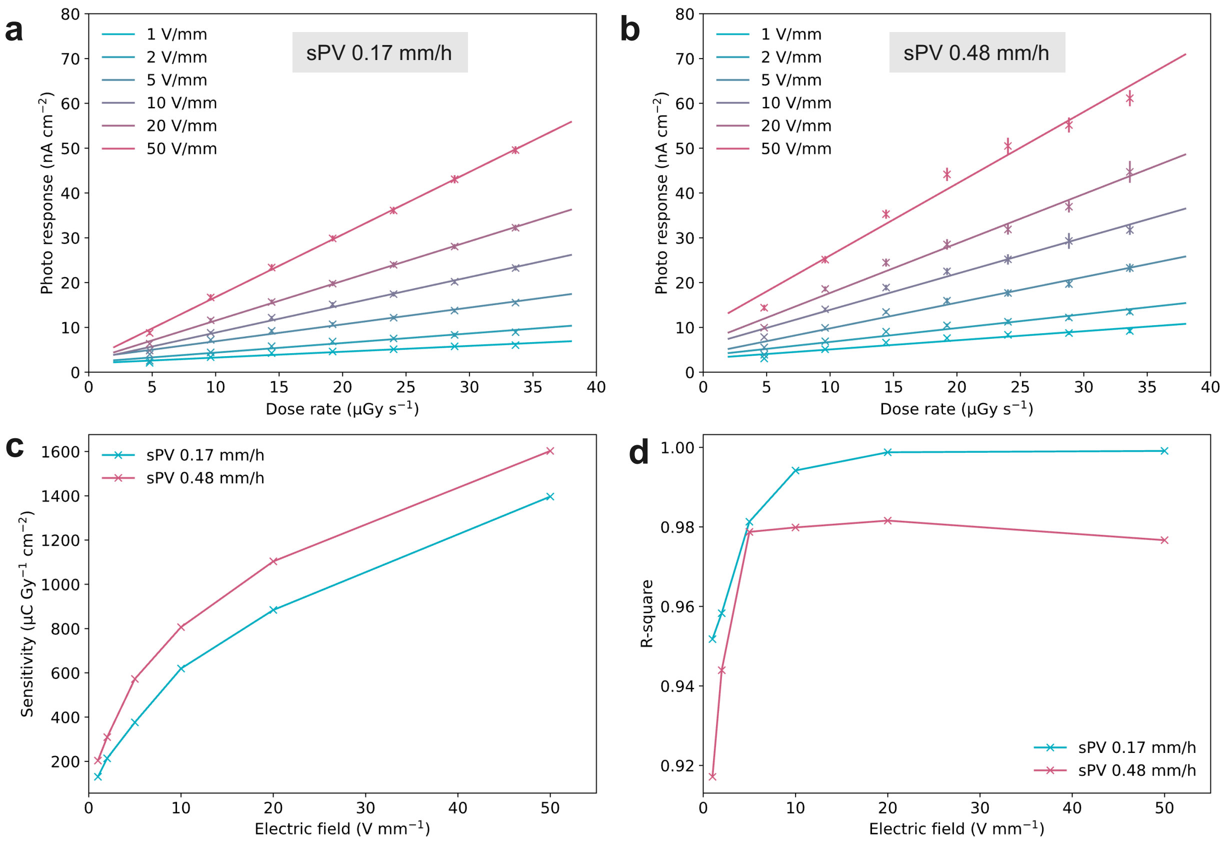

Fig. S26| a–b. Photoresponses to X-rays of sPV-0.17 (a) and sPV-0.48 devices at different biases of 1–50 V mm^{-1}. The '×' points are the measured data. The lines show the linear fitting. **c.** X-ray detection sensitivity derived from the slope of the linear fitting in panels **a** and **b**. **d.** R-square values for the fitting.

Table S1| The full-width half at maximum of the rocking curves of $MAPbBr_3$ single crystals

Method	Growth temp. (ºC)	Crystal plane	FWHM (arcsec)	Ref.
ITC	85	(100)	25.6	1
AVC	38	(100)	60.2	[5]
LDSC	40	(100)	34.6	[6]
LDSC	60	(100)	48.6	[6]
ITC	-	(100)	108.0	[6]
ITC	100	(100)	284.4	[8]
LTGC	60	(100)	68.4	[8]
ITC	100	(100)	406.8	[9]
LTGC	60	(100)	68.4	[10]
CGC	35	(220)	64.8	[10]
ITC	90	(100)	65.9	[11]
LDSC	RT	(100)	58.7	[12]
ITC	110	N/A	61.6	10
SE	34	(200)	19.3	11
FRC	40	(100)	15.3	This work
FRC	40	(200)	14.0	This work
FRC	40	(300)	11.8	This work
FRC	40	(400)	13.8	This work

*ITC: inverse temperature crystallization, AVC: antisolvent vapor-assisted crystallization, LDSC: liquid diffused separation induced crystallization, LTGC: low temperature gradient crystallization, CGC: counterdiffusion-in-gel crystallization, SE: solvent evaporation, FRC: flux-regulated crystallization

References

1. Amari, S., Verilhac, J.-M., Gros D'Aillon, E., Ibanez, A. & Zaccaro, J. Optimization of the Growth Conditions for High Quality $CH_3NH_3PbBr_3$ Hybrid Perovskite Single Crystals. *Cryst. Growth Des.* **20**, 1665–1672 (2020).

2. Wenger, B. *et al.* Consolidation of the optoelectronic properties of $CH_3NH_3PbBr_3$ perovskite single crystals. *Nat. Commun.* **8**, 590 (2017).

3. Xing, J. *et al.* Thickness-dependent carrier lifetime and mobility for $MAPbBr_3$ single crystals. *Materials Today Physics* **14**, 100240 (2020).

4. Bazalova-Carter, M. & Esplen, N. On the capabilities of conventional x-ray tubes to deliver ultra-high (FLASH) dose rates. *Med. Phys.* **46**, 5690–5695 (2019).

5. Zhang, L. *et al.* Anisotropic Performance of High-Quality $MAPbBr_3$ Single-Crystal Wafers. *ACS Appl. Mater. Interfaces* **12**, 51616–51627 (2020).

6. Zhang, Z. *et al.* Controllable Growth of High Quality $MAPbX_3$ Perovskite Single Crystals for X-ray Detection. *ACS Appl. Electron. Mater.* (2022) doi:10.1021/acsaelm.2c01424.

7. Liu, Y. *et al.* Low-temperature-gradient crystallization for multi-inch high-quality perovskite single crystals for record performance photodetectors. *Mater. Today* **22**, 67–75 (2019).

8. Liu, Y. *et al.* A 1300 mm^2 Ultrahigh-Performance Digital Imaging Assembly using High-Quality Perovskite Single Crystals. *Adv. Mater.* **30**, 1707314 (2018).

9. Selivanov, N. I., Murzin, A. O., Yudin, V. I., Kapitonov, Y. V. & Emeline, A. V. Counterdiffusion-in-gel growth of high optical and crystal quality $MAPbX_3$ ($MA = CH_3NH_3^+$, $X = I^-$, Br^-) lead-halide perovskite single crystals. *CrystEngComm* **24**, 2976–2981 (2022).

10. Yao, F. *et al.* Room-temperature liquid diffused separation induced crystallization for high-quality perovskite single crystals. *Nat. Commun.* **11**, 1194 (2020).

11. Cho, Y. *et al.* High speed growth of $MAPbBr_3$ single crystals via low-temperature inverting solubility: enhancement of mobility and trap density for photodetector applications. *Nanoscale* **13**, 8275–8282 (2021).

12. Li, W. *et al.* Fine-control-valve of halide perovskite single crystal quality for high performance X-ray detection. *Sci Bull. Fac. Agric. Kyushu Univ.* **66**, 2199–2206 (2021).
